# Cu^2+^–Assisted Synthesis of Ultrasharp and Sub-10 nm Gold Nanostars. Applications in Catalysis, Sensing, and Photothermia

**DOI:** 10.1021/acsanm.4c03310

**Published:** 2024-08-15

**Authors:** Esraa Samy Abu Serea, Leixuri B. Berganza, Senentxu Lanceros-Méndez, Javier Reguera

**Affiliations:** † BCMaterials, Basque Center for Materials, Applications, and Nanostructures, UPV/EHU Science Park, Leioa 48940, Spain; ‡ Ikerbasque, Basque Foundation for Science, Bilbao 48009 Bilbao, Spain; § Department Condensed Matter Physics, University of Valladolid, Bioforge, Pso. de Belén 19, 47011 Valladolid, Spain

**Keywords:** gold nanostars, anisotropic nanoparticles, tip sharpening, hyperthermia, catalysis, SERS

## Abstract

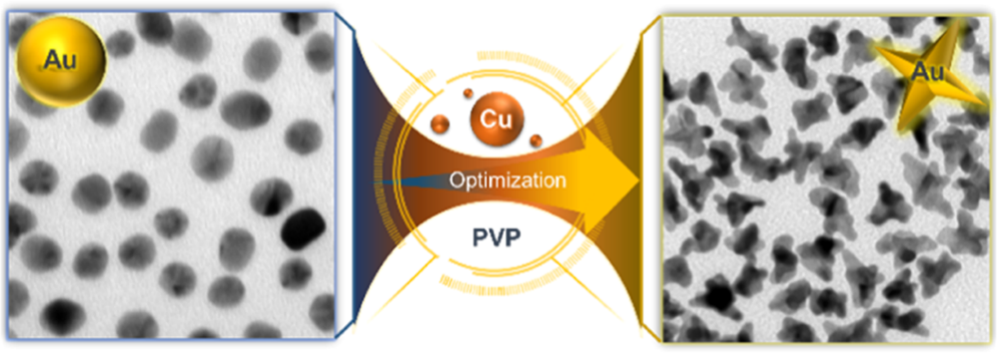

Gold nanostars have shown enormous potential as the main enablers of advanced applications ranging from biomedicine to sensing or catalysis. Their unique anisotropic structure featuring sharp spikes that grow from a central core offers enhanced optical capabilities and spectral tunability. Although several synthesis methods yield NSs of different morphologies and sizes up to several hundred nanometers, obtaining small NSs, while maintaining their plasmonic properties in the near-infrared, has proven challenging and elusive. Here, we show that Cu^2+^ addition during NS synthesis in polyvinylpyrrolidone/dimethylformamide generates more crystallographic defects and promotes the directional growth, giving rise to NSs with a larger number of much sharper spikes. They are also formed at smaller volumes, enabling the generation of ultrasmall nanostars, with a volume as small as 421 nm^3^ (i.e., 9.2 nm of volume-equivalent diameter), while maintaining a plasmon resonance in the near-infrared. To this end, we systematically evaluate the influence of synthesis parameters on the nanostar size and optical characteristics and demonstrate their properties for applications in catalysis, surface-enhanced Raman spectroscopy sensing, and hyperthermia. The ultrasmall nanostars show excellent attributes in all of them, leveraging their small size to enhance properties related to a higher surface-to-volume ratio or colloidal diffusivity.

## 1 Introduction

Multibranched gold (Au) nanoparticles, also known as Au nanostars (NSs), nanoflowers, or nanourchins, are prominent examples of plasmonic nanostructures.^1−3^ They consist of spikes (also called tips) that protrude from a central nanoparticle body. This unique design produces a very intense enhancement of the electromagnetic field at the tip of the spikes (intrinsic hotspots) as a result of the localized surface plasmon resonance (LSPR), and the lightning-rod effect.^4^ In addition, Au NSs show LSPR tunability from the visible to the near-infrared (NIR) region by altering their spikes aspect ratio, which, together with their high intense hot spots, makes them outstanding candidates in several optical and plasmon-related applications.^5,6^ Among those applications, Au NSs are key enablers in imaging, sensing, drug delivery, catalysis, surface-enhanced Raman scattering (SERS), or photothermal therapy applications.^7−9^ For instance, in catalysis, the high surface area and sharp tips of NSs provide active sites that enhance reaction efficiency and selectivity compared to other nanostructures.^1,3^ Furthermore, this unique morphology significantly increases sensitivity in SERS sensing applications, allowing for lower detection limits and making NSs highly effective for detecting trace amounts of analytes.^4^ Additionally, NSs are promising for hyperthermia applications due to their ability to absorb light efficiently and convert it into heat.^8^ Recent enhancement in the synthesis of various morphologies and sizes of NSs has further expanded their potential applications by combining catalytic, sensing, and hyperthermia capabilities.

Several synthesis methods have been developed to produce NSs with different morphologies, in terms of spike length, number of spikes, symmetry, or spike sharpness. They are typically synthesized by wet chemical methods that involve a solvent, a gold salt (typically chloroauric acid), a reducing agent, a shape-directing agent, and a stabilizing agent. In some cases, some reagents can have more than one function (for instance, reducing, stabilizing, and shape directing). They are typically classified according to the type of reagents used for those functions, especially the shape-directing agent. The shape-directing agents, also related to the symmetry breaking in the nanoparticle growth, are necessary to control the spike growth with different morphologies. Among them there are surfactants such as cetyltrimethylammonium bromide or polymers such as polyvinylpyrrolidone (PVP). Some metal ions, like Ag or Cu, have also been shown to be excellent shape-directing agents.^2,10−12^ Moreover, Au NSs can be classified between seedless and seed-mediated-growth synthesis methods. In this last case, small gold nanoparticles (seeds) are added to the reaction to avoid the nucleation step, having only crystal growth and producing highly uniform NSs in terms of size distribution.

One example of a seedless method is the one that uses Good’s buffers such as 4-(2-hydroxyethyl)-1-piperazineethanesulfonic acid (HEPES), 2-(2-hydroxyethyl)-1-piperazinepropanesulfonic acid (EPPS), and 3-(*N*-morpholino)propanesulfonic acid (MOPS).^13,14^ In these cases, the buffer performs the functions of shape-directing, reducing, and stabilizing agents, and the NSs show asymmetric spikes. They offer certain tunability, with limited control in the uniformity and size range between approximately 40 and 200 nm. Hu et al., for example, described the fabrication of small 29 nm Au NSs by a seedless synthesis based on HEPES buffers. By changing the HEPES and HAuCl_4_, the authors demonstrated that the high proportion of HEPES to HAuCl_4_ leads to a red shift of the main LSPR peak of the Au NSs.^15^ Similarly to seedless, the NSs obtained from seed-mediated-growth methods can be achieved with a wide range of sizes up to several hundreds of nanometers (Table S1). One example of this type of synthesis is the surfactant-free Au NSs, which involves ascorbic acid as a reducing agent and silver ions as shape-directing agents. Theodorou et al., for instance, synthesized relatively small Au NSs approximately 47 nm in size using a seed-mediated-silver-assisted method. By controlling synthesis parameters, authors achieved variations in the number and sharpness of their spikes. Larger Au NSs displayed more spikes with sharper tips, while smaller Au NSs exhibited fewer spikes and more rounded tips.^16^ Other examples of seed-mediated-growth methods are the ones using PVP in dimethylformamide (DMF), which acts as a reducing, shape-directing, and stabilizing agent.^17−20^ Barbosa et al., for instance, introduced a PVP/DMF synthesis method yielding multibranched NSs with narrow tip plasmon resonance bands. They observed that for small [HAuCl_4_]/[seed] molar ratio values, they obtained some of the smallest types of NSs, still limited to sizes typically bigger than 20 nm.^21^

While achieving large sizes seems relatively simple, the pursuit of achieving small sizes below 10 nm while interacting in the NIR poses a considerable challenge and seems elusive (see Table S1 for typical sizes). The limitation in the minimum size relies on the needed amount of Au for breaking the symmetry that makes the spikes grow in different directions as well as the needed amount of Au to create the spikes with enough aspect ratio to produce the plasmonic red shift needed for applications in the NIR.^22,23^ Moreover, controlling the sharpness of the NS spikes, needed for forming highly intense hot spots, presents challenges similar to those observed in obtaining small NSs. This sharpness of NS spikes typically extends to a range of 2–13 nm in radius of curvature (ROC) (see Table S1), with very few cases in the proximity of 2 nm. Therefore, understanding the synthesis methods and factors influencing the limits in size and sharpness is essential for optimizing Au NSs for specific applications.

Despite its challenging synthesis, small NSs exhibit a range of characteristics that make them desirable for many applications, including, among others, (1) a better penetration into tissues and cells, (2) a higher diffusion coefficient for better mixing and distribution, (3) long-term circulation stability and biokinetics, and (4) higher surface-to-volume ratio for drug and functionality loading or catalytic processes. Moreover, the small size requires smaller and sharper spikes, generating the formation of more intense hotspots, which would make the NSs excellent candidates in SERS sensing and imaging.^24−29^

Here, we show how adding Cu^2+^ to the PVP/DMF synthesis produces NSs with extremely sharp spikes, the formation of which occurs at early times in the growth process. We have taken advantage of this characteristic to obtain unprecedently small NSs, hereafter called ultrasmall nanostars (USNSs). For that, we devoted our efforts to optimizing their synthesis parameters (seed injecting time, PVP, Cu^2+^, and seed relative concentrations) to obtain smaller sizes while still obtaining plasmonic properties in the NIR regions. Finally, we have demonstrated their potential capabilities in applications such as catalysis of nitrophenol reduction, SERS sensing in colloidal solutions, and plasmonic photothermia.

## 2 Results and Discussion

### 2.1 Study of Synthesis Parameters of USNSs

#### 2.1.1 Effect of Cu^2+^ on the Spikes’ Radius of Curvature

**Figure 1:**
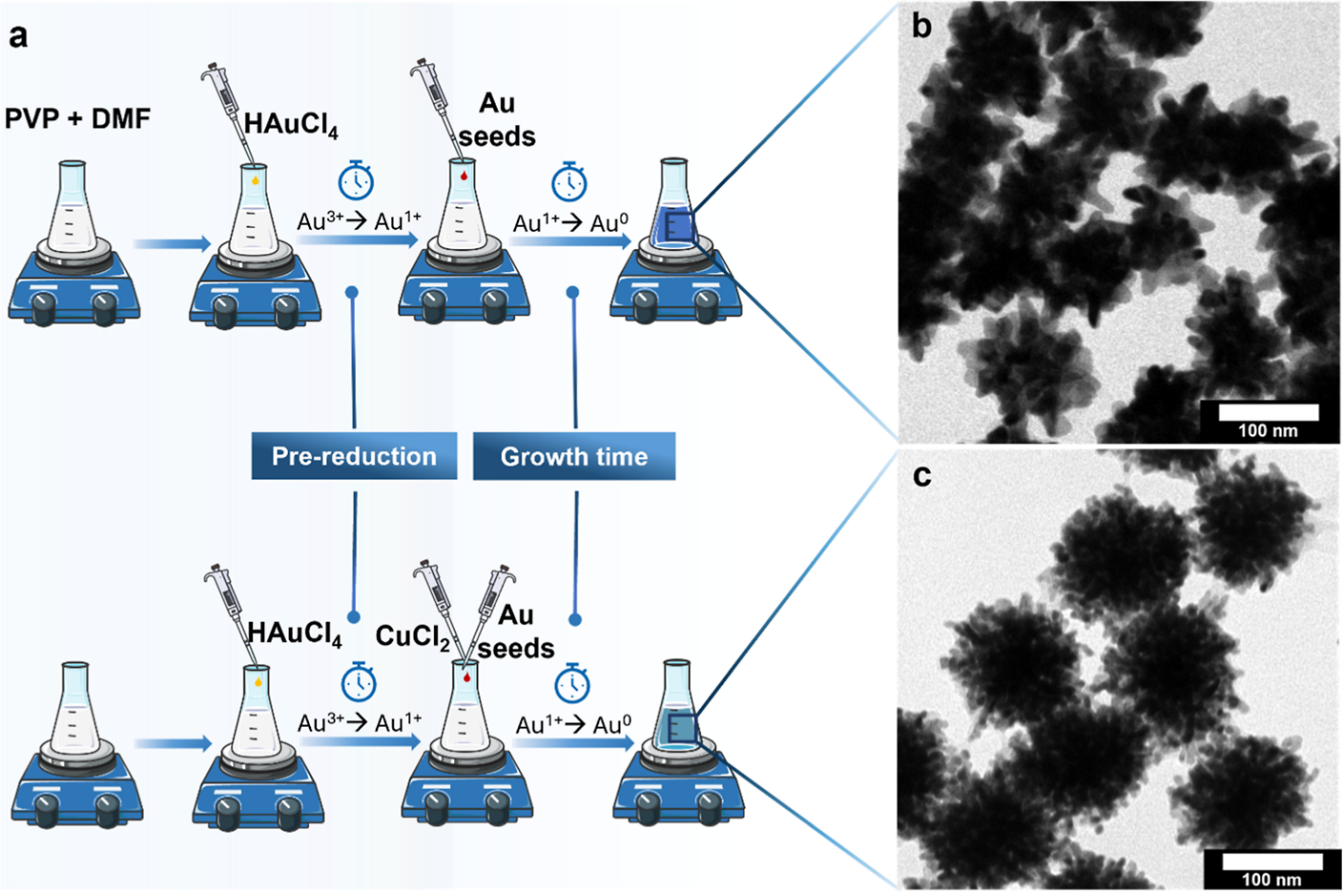
(a) Scheme illustrating the synthesis process of Au NSs with and without Cu^2+^ addition. (b,c) TEM images of two different morphologies of Au NSs, which are synthesized without (b) and with (c) Cu^2+^ precursor, respectively.

We have found that the introduction of Cu^2+^ during the reduction of Au^3+^ in the PVP/DMF synthesis of Au NSs highly influences their morphological characteristics and the structural growth at the spikes, therefore modifying their optical properties. Figure 1a shows schematically the synthesis process of Au NSs through the PVP/DMF seed-mediated-growth wet chemical method without and with the addition of Cu^2+^ (full description in the Supporting Information). Figure 1b,c shows the transmission electron microscopy (TEM) images corresponding to the two types of NSs under similar conditions with the same average nanoparticle volume and a diameter of around 100 nm (124.9 ± 6.14 and 101.2 ± 7.46 nm, for area-equivalent diameter respectively).

At first sight, the two types of NSs show completely different morphologies. The NSs without Cu^2+^, the standard synthesis, present a lower number of spikes, which are larger, thicker, and straighter (Figure 1b). On the contrary, the NSs with Cu^2+^ show a much higher number of spikes, which are considerably thinner, sharper, and more irregular in shape (Figure 1c). The sharpness of those spikes can be easily measured using the ROC. They present a ROC value of 3.24 ± 0.80 nm for the standard NSs (size distribution can be found in Figure S1) and 1.34 ± 0.30 nm for the NSs with Cu^2+^, which, to the best of our knowledge, is the smallest of all reported NSs in the literature (Table S1). The decrease in ROC and the higher irregularity of the spikes could be attributed to the generation of defects on the crystal facets during the growth process, which breaks the fcc symmetry of the Au lattice and promotes the growth in new directions, aided by the use of two simultaneous shape-directing agents PVP and Cu^2+^. Further, the tunability achieved by the introduction of Cu^2+^ provides new possibilities to tune the synthesis conditions for size and morphological control.

#### 2.1.2 Influence of Reaction Parameters on the Synthesis of USNSs

**Figure 2:**
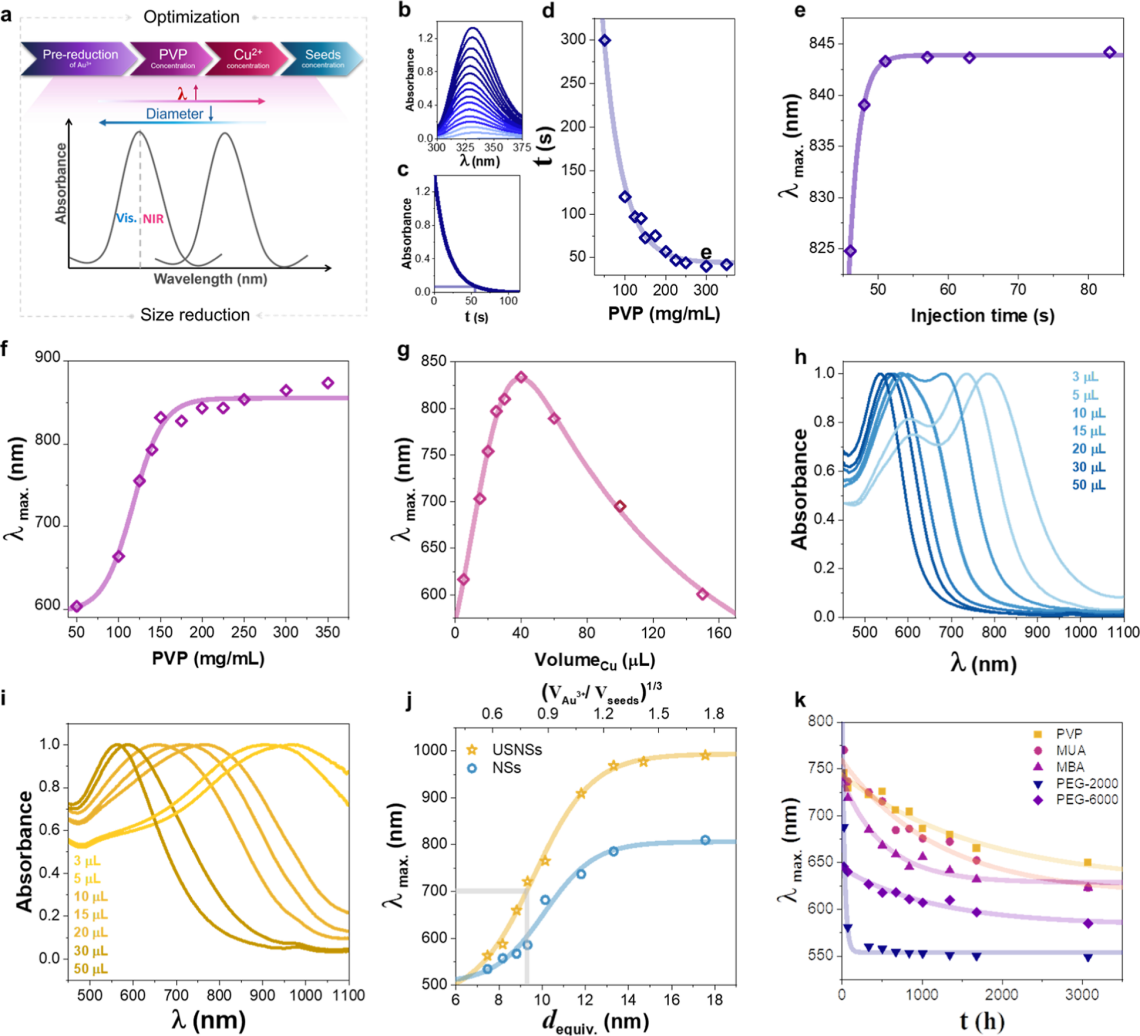
(a) Step-by-step optimization of reaction parameters (injection time, and concentration of PVP, Cu^2+^, and seeds) with the first three steps producing a red shift and no size change, and the last one decreasing the size by using the gained shift. (b) UV–vis spectra of the reduction of Au^3+^ to Au^1+^ at 200 mg/mL PVP concentration. (c) Absorbance at 340 nm vs time for the spectra in (b). (d) Time for reduction up to 4% of Au^3+^ (96% of Au^1+^) as a function of PVP concentration. (e) Effect of injection time: Wavelength at maximum absorbance (λ_max_) as a function of prereduction time (time for seed injection). (f) Variation of λ_max_ with the PVP concentration tuning. (g) Variation of λ_max_ with the change of Cu^2+^ added volume. (h–j) Normalized UV–vis spectra for seed volume variation of (h) NSs without Cu^2+^ and (i) USNSs with Cu^2+^. (j) λ_max_ variation as a function of the volume ratio (*V*_Au_3+__/*V*_seeds_)^1/3^ and equivalent diameter. (k) Long time λ_max_ shift of USNSs (reshaping) after functionalization with different stabilizing ligand molecules.

One clear consequence of the thinner and sharper spikes is that a much smaller amount of Au is needed for their growth; therefore, in principle, smaller NSs can be obtained. Moved by this idea, we have studied the influence of Cu^2+^ on the synthesis of ultrasmall NSs (USNSs). To establish a definition of NSs and to differentiate them from a slightly deformed nanoparticle or embryonic NSs, we have used as an indicator the amount of red shift of the LSPR spike-related band with respect to the dipolar band of the equivalent spherical nanoparticles. Here, we selected 700 nm as a wavelength threshold for the nanoparticles to be considered as NSs. This threshold was chosen given the importance of working in the NIR region for many applications. To study how the different synthesis parameters allow for smaller NSs, the initial synthesis was performed starting with standard reported conditions, using small-size seeds of 3.7 nm and with the smallest size that gave an LSPR higher than 700 nm. Then, the variation of the wavelength of maximum absorbance (λ_max_) with respect to each parameter was followed. We know that for small NS sizes, the bigger the NSs, the higher the λ_max_ becomes, which means we can use this parameter as an indicator of how we can reduce the size of the NSs (Figure 2a). In other words, the more we can red shift λ_max_ by varying one of the parameters that do not change the size of the NSs, the smaller we can make the NSs later by varying the ratio of seed and growth solution. Finally, after the different synthesis parameters were optimized, the added seed volume for a fixed growing solution was tuned to obtain the smallest USNSs still showing the LSPR band above 700 nm.

#### 2.1.3 Effect of Prereduction Kinetics of Au

As previously shown in standard NSs, prereduction of Au^3+^ to Au^1+^ before the addition of seeds in the synthesis is highly beneficial for the formation of uniform NSs as it prevents the oxidation of the Au seeds by Au^3+^ ions.^21^ PVP reduction kinetics were performed, followed by UV–vis by measuring the decrease of absorbance at 340 nm, corresponding to Au^3+^ absorption. Figure 2b shows an example corresponding to a concentration of PVP of 200 mg/mL. The absorbance exponentially decreases over time as Au^3+^ is transformed into Au^1+^ (Figure 2c). If much longer times are applied (for instance to obtain 100% transformation), the appearance of Au^0^ would produce an increase of absorbance at this wavelength, which should be avoided as it could generate the formation of new nuclei and hamper the synthesis of uniform NSs. As the prereduction time depends on the concentration of PVP, similar experiments were performed by varying this concentration. Figure 2d shows the time to reduce 96% of the Au^3+^ at different PVP concentrations and how an increase in PVP increases the kinetics of the reaction.

Figure 2e shows the wavelength of maximum absorbance (λ_max_) as a function of time for the seed’s injection, corresponding to the range of reduction of Au^3+^ between 93 and 99%. The graph shows a monotonic increase up to a threshold time, which takes place around 96% of prereduction and a plateau after that percentage. Similar behavior was observed for other PVP concentrations. Therefore, a time for injection corresponding to 96% reduction of Au^3+^ produced the best results to obtain USNSs and avoided unwanted nucleation. Note here that the prereduction time does not change the size of the NSs, but this gained red shift will be used later to decrease the size of the NSs by varying the volume of added seeds (Section 2.1.6).

#### 2.1.4 Effect of PVP Concentration

PVP plays multiple functions in the seed-mediated synthesis of NSs: it generates an amphiphilic coating that provides colloidal stability in solvents that go from relatively apolar solvents, such as chloroform, to polar solvents such as water or cell media; it acts as a shape-directing agent as it exhibits a preference for adsorption onto {111} planes of the Au, hence preventing the further growth in these directions and promoting the anisotropic growth of nanoparticles in the form of NSs;^17,30^ finally, it acts as the reducing agent of Au^3+^ and consequently affects the NS formation kinetics. On the whole, the modification of its concentration should have a profound effect on tuning the optical features of USNSs.

Figure 2f shows the variation of λ_max_ as a function of the PVP concentration. The plasmonic red shift quickly increases with PVP concentration up to around 150 mg/mL followed by a small steady increase after this value. For very high concentrations, the solution is quite viscous and takes a long time to dissolve; therefore, a compromise PVP concentration of 200 mg/mL was chosen to obtain smaller USNSs. Like in the previous section, the PVP does not change the final size of the NSs, but the positive red shift obtained here will be used to obtain smaller sizes in the last section of optimization. Note here that some differences, in terms of reactivity, appear depending on the PVP used (molecular weight, brand, and batch), which could slightly vary the optimal PVP concentration. For instance, we achieved the smallest size using TCI brand and a molecular weight of 10 kg/mol (as we will see later up to 9.2 nm), while using a Sigma-Aldrich the smallest size was 14.6 nm for one of the used batches and no reduction for a second batch (see experimental part). This highlights the importance of selecting the correct PVP in Au NSs synthesis, as it has a critical impact when very small NSs are required.

#### 2.1.5 Effect of Cu^2+^ Concentration

The same protocol was applied to assess the influence of the added volume of CuCl_2_·2H_2_O on USNS formation (5–150 μL at 5 mM, for a fixed reaction volume). For small quantities of Cu^2+^, λ_max_ rapidly increased with increasing Cu^2+^ content, until it reached a threshold volume at 40 μL. Then, λ_max_ rapidly dropped to lower values (Figure 2g). This can be rationalized as follows; the incorporation of small amounts of Cu^2+^ into the growth process of USNSs helps in the creation of defects that break the crystal symmetry and promote the growth in certain directions; however, when too much Cu^2+^ is added, the NPs start behaving as a homogeneous alloy suppressing the directionality effect. The addition of small quantities of Cu^2+^, apart from giving rise to smaller and sharper spikes, can be advantageously used for easily tuning the plasmonic properties without the need to change the NS size. As seen in Figure 2g, for quantities below 30 μL, an almost linear variation is obtained.

We selected 40 μL as the optimum value to produce the highest red shift possible. Although adding Cu could increase the size of the NSs, as we will see below, the little incorporation of Cu in the final NS makes the size of the NSs almost independent of this parameter; therefore, we can assume that a red shift in the plasmonic band will allow us to obtain smaller NSs in the next section by increasing the volume of seeds at a given growth volume.

#### 2.1.6 Tuning the Size by Changing Seeds to Growth Volume Ratio

**Figure 3:**
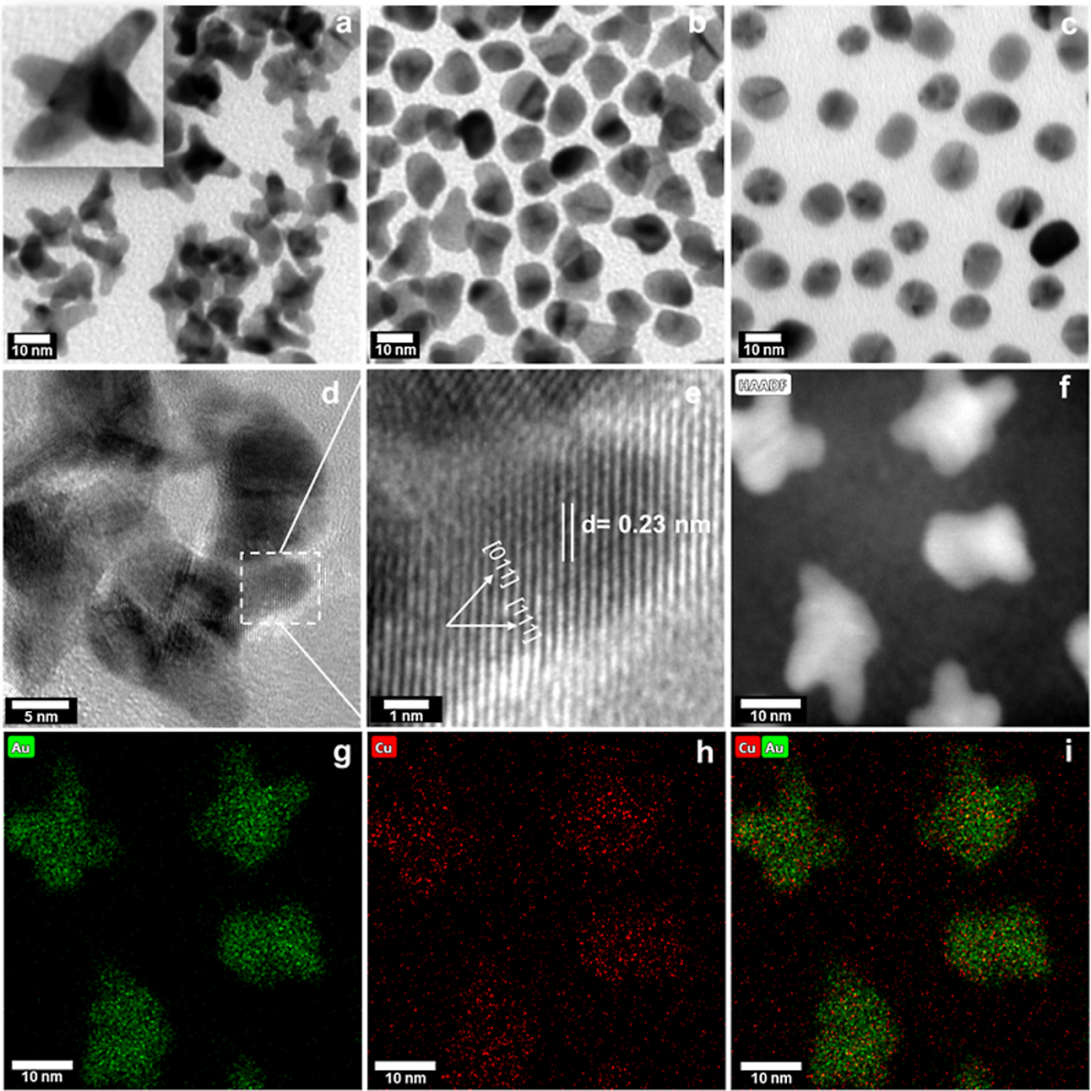
TEM images of different types of nanoparticles with the same average volume and three different morphologies corresponding to (a) USNSs with λ_max_ around 700 nm (inset magnified image of single USNS), (b) NSs, obtained with the optimized parameters except the addition of Cu^2+^, and (c) equivalent NSphs used to calibrate the nanoparticle volume. (d,e) HR–TEM images of USNSs with the TEM fringes of (e) corresponding to the (111) planes. (f–i) Elemental mapping images of USNSs structure: (f) STEM–HAADF image of the selected area. (g–i) STEM–EDX maps of Au alone (green), Cu alone (red), and overlap map of Au and Cu.

Since the synthesis method is a seed-mediated-growth process, the final USNSs are always highly monodisperse in size. Moreover, this size can be perfectly tuned by adjusting the ratio between the added seeds and the amount of Au precursor in the growth solution (*V*_Au_3+__/*V*_seeds_ ratio). It is also evidenced that the higher the amount of Au seeds introduced in the reaction, the smaller the USNSs that are produced. Figure 2h,i shows how the spectrum changes as we vary the added volume of seeds, for the optimized conditions mentioned above, without and with the addition of Cu^2+^, respectively. For those sizes, the NSs with Cu show broader bands that extend more toward the NIR region. Figure 2j summarizes the degree of the λ_max_ red shift, from the previous two graphs, versus the ratio of *V*_Au_3+__/*V*_seeds_ for the tested ratios. The cubic root was used in this case since this value is proportional to the sphere-equivalent diameter of the nanoparticles, d_equiv_. To precisely determine the size of the different NSs, equivalent spherical nanoparticles, hereafter called nanospheres (NSphs), were synthesized by using the same volumes of growth solution and seeds but performing the synthesis at a much higher temperature, which rounded the nanoparticles, obtaining almost spherical morphologies. A calibration line was then obtained between *d*_equiv_ and (*V*_Au_3+__/*V*_seeds_)^1/3^ for those nanospheres (Figure S3) and used in the second *x*-axis for the Au NSs in Figure 2j. At very low *d*_equiv_, the NS synthesis gave rise to a low λ_max_ indicative of quasi-spherical nanoparticles. After a certain *d*_equiv_, the NSs spikes start forming, and a new band, corresponding to the spikes’ hybrid dipolar plasmonic mode, appears. The spikes’ aspect ratio increases rapidly up to a certain nanoparticle size, therefore exhibiting an acute λ_max_ increase. After the spikes are well formed, a much slower increase vs size takes place. For comparison, for the NSs synthesized without Cu^2+^ (Figure 2j, blue), the volume required to start forming the NS spikes, even for this optimized protocol, was substantially bigger, and therefore the size to have a plasmonic band in the NIR. The smallest USNSs synthesized while presenting a band in the NIR (λ_max_ ≥ 700 nm) was obtained from the fitted function (eq S1) and showed a volume as small as 421 nm^3^ and a sphere-equivalent diameter *d*_equiv_ = 9.2 nm, which, to the best of our knowledge, is unprecedented for the synthesis of NSs. On the other hand, we have observed that in the optimization we performed but without Cu^2+^, we also obtained a relatively small NS volume corresponding to 697 nm^3^, which is still much bigger than with Cu^2+^, and a corresponding *d*_equiv_ = 11 nm. It is clear that at those small sizes, the difference in λ_max_ should be reflected in the morphology, as shown in the TEM images of Figure 3a–c. The as-prepared NSs show high uniformity given by the seed-mediated growth method and the absence of homogeneous nucleation (Figure S4). Figure 3a corresponds to USNSs with λ_max_ slightly above 700 nm, presenting well-formed USNSs with 3–5 spikes per USNS. On the contrary, for the optimized synthesis of the NSs formed without the addition Cu^2+^ (Figure 3b), only irregular nanoparticles with a structure of slightly deformed spheres with initial broad protuberances appear. Finally, for a comparison, Figure 3c shows the NSphs with the same volume that the NSs used to quantify the NSs volume and equivalent diameter.

### 2.2 USNSs’ Structural Properties

#### 2.2.1 USNSs’ Reshaping

Au NSs are known to reshape, given time and energy, like any other Au nanoparticle with anisotropic shapes tending to spheres.^14,31^ We have observed that the blue shift due to reshaping of USNSs, given the small size and high sharpness of the spikes, occurs more rapidly than with standard NSs. One known way to hamper this is with the use of coating agents that, apart from being responsible for the colloidal stability, hinder the Au atoms’ movement and therefore the reshaping.^32^ To acquire a better understanding of the coating effect, different functionalizations with ligand molecules were compared by monitoring the shifting of LSPR bands. Figure 2k shows the λ_max_ of USNSs along ∼4 months after functionalization. For all capping molecules, the highest blue shift took place in the first day, while a much slower reshaping was produced after that. The native coating after the synthesis (PVP) revealed long-term stability as indicated by a low blue shift of the LSPR bands (λ_max_ ∼ 745–680 nm). However, for USNSs functionalized with thiolated molecules, large differences appear. 11-Mercaptoundecanoic acid (MUA) showed the highest shape stability, followed by 4-mercaptobenzoic acid (MBA). On the other hand, thiolated polyethylene glycol (PEG-SH), which is one of the most used capping ligands, presented a lower shape stability. Interestingly, increasing its molecular weight, which typically improves colloidal stability, improved also the shape stability.

#### 2.2.2 USNSs’ Structure Characterization

ICP analysis of the USNSs revealed that the reduction of Au had a practically 100% yield. However, for the case of the reduction of Cu^2+^ to Cu and its incorporation in the NSs, less than 10% was incorporated ([Au^3+^]/[Cu^2+^] = 1.46 used in the reaction vs [Au]/[Cu] = 26.6 measured in the USNSs by ICP). The observed ultralow Cu content can be attributed to the lower standard reduction potential of Cu^2+^ of 0.34 V versus the ones of Au^3+^ or Au^1+^ of around 1 V (see Supporting Information) and therefore a lower tendency to undergo reduction compared to Au ions under similar conditions. Finally, the small quantity of reduced Cu incorporated in the USNS appears homogeneously distributed through the USNS as observed in the STEM–EDX mapping (Figure 3d–i). Selected-area EDX spectrum can be seen in Figure S5 showing the main presence of Au (98.05 wt %) together with the smaller quantity of Cu (1.95 wt %) in line with the results observed in ICP.

The lattice structure of the USNSs was studied by X-ray Diffraction (XRD) and high-resolution TEM (HR-TEM). XRD diffractogram (Figure S6) showed high crystallinity with the expected Au fcc lattice, with three clear peaks identified as (110), (200), and (220). The peak position indicates a lattice parameter of *a* = 4.06 Å, which matches the lattice parameter of bulk Au (*a* = 4.07 Å) and Au nanoparticles for this range of sizes.^49^ This result agrees with the low incorporation of Cu atoms inside of the USNSs and therefore the negligible change in the lattice. Scherrer’s equation was used to determine the crystallite sizes (eq S2). The crystallite average size corresponded to *d* = 7.86 nm, which agrees with the polycrystalline nature of the NSs, while exhibiting small monocrystalline domains similar to the USNSs’ spike sizes. The lattice structure was also visualized in HR-TEM micrographs (Figure 3d,e). Multitwin boundaries are present at the USNSs core as shown in Figure 3d. Figure 3e shows the single-crystal structure of a USNS spike with interplanar distances of 0.23 nm, corresponding to easily visible (111) planes, which agrees with the [011] growing direction previously observed for standard NSs obtained by the PVP/DMF method.^18,33^ For bigger NSs (Figure 1b), the spikes show some irregularities indicative of extra crystallographic defects appearing at larger growths.

Anisotropic growth of NSs is governed by a complex interplay of multiple factors.^34^ In our USNSs, the tip growth can be driven as a combined effect of Cu^2+^,^35,36^ along with other shape-directing agents (the capping agent PVP) during the growth process.^37^ As it has been reported for pentacle nanocrystals of Au–Cu alloys synthesized with different capping agents, the growing direction is dominated mainly by the selection of the capping agent. The surface adsorption with the capping agent drives a preferential adsorption on {111} planes, which are energetically favorable sites, leading to the formation of defects and multitwinned Au nanoparticles and a growth in the [011] direction.^11,37^ The presence of the second precursor (Cu^2+^) was crucial for inducing tip growth on less common exposing facets along certain crystallographic directions. Cu^2+^ ions can replace Au atoms within the crystal lattice and/or occupy interstitial sites between gold atoms and affects the spacing between neighbor atoms, which lead to lattice strain (lattice mismatch) and distortions. This strain modifies the surface energy and reactivity, which accelerates anisotropic growth.^38−40^ Cu^2+^ atoms can deposit along the [001] direction, affecting the arrangement of Au atoms at the tips, creating sharper edges but with less control over the shape.^41^ As reported in pentacle nanocrystals, the increase of Cu^2+^ has been seen to increase the growth in the direction of the planes, with less preferential adsorption of the capping agent producing a higher elongation of the crystal arms as the quantity is increased.^11,42^ In addition, the underpotential deposition of Cu, which has been well described on flat surfaces of Au, could accelerate the growth in certain directions, in this case, accelerating the growth in the spike’s directions; however, as has been pointed out,^42^ this would create a nonhomogenous distribution of the Cu inside the NS. Although this is not our case, as we observed a homogeneous distribution, this could be due to a migration of Cu atoms inside the lattice as the NS is formed. On the whole, we have seen that by modulating the kinetic parameters in each step of optimization, we achieved precise control over the structure and enabled the formation of sub-10 nm USNSs. The morphology and plasmonic properties of these small stars exhibit notable differences from previously reported NSs, in which each NS featured rounded tips and poor optical properties at the NIR for those small NS sizes.^15,18,21,43,44^

## 3 Applications of USNSs

Au NSs have been used in a wide range of applications. Here, we have explored the use of USNSs in some of them, including the catalytic reduction of 4-nitrophenol, SERS, and plasmonic hyperthermia therapy (photothermia). Note here that, despite their performance in those applications, the reduced size of the USNSs offers additional features to overcome more complex application necessities, such as higher diffusion coefficients, higher circulating time in biological media, more penetrability in tissues, or a higher surface-to-volume ratio to improve reactivity and nanoparticle loading.

### 3.1 Catalytic Reduction of 4-Nitrophenol

**Figure 4:**
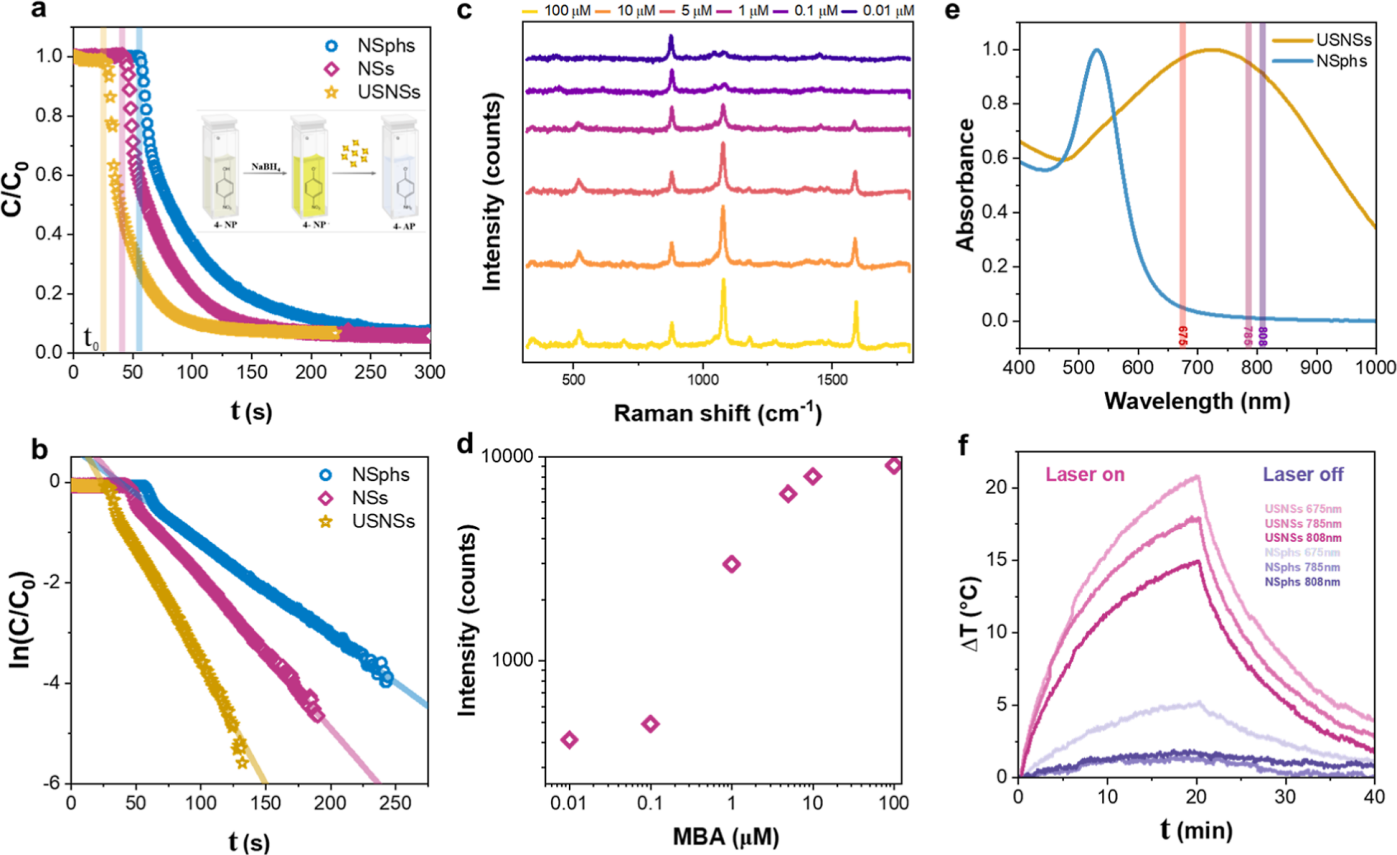
(a) Catalytic reduction of 4-NP to 4-AP using USNSs, NSs, and NSphs as catalysts. The inset shows the corresponding catalytic chemical steps of the reaction and their respective colors. (b) Plots of ln(*C*/*C*_0_) versus time for USNSs, NSs, and NSphs. (c,d) SERS measurements of the model molecule 4-MBA. (c) SERS spectra for varying concentrations of 4-MBA showing two intense peaks at Raman shifts of 1080 and 1587 cm^–1^. (d) Intensity of the peak at 1080 cm^–1^ as a function of the 4-MBA concentration. (e) UV–vis extinction spectra of USNSs and NSphs used for photothermia with respect to the laser wavelengths. (f) Δ*T* thermogram of an aqueous dispersion (1 mL, 0.2 mM) of USNSs and NSphs exposed to laser illumination (*I* = 0.33 W/cm^2^, and λ = 675, 785, 808 nm) in an on–off experiment.

Here, the catalytic activity of USNSs was evaluated by using the reduction of 4-nitrophenol (4-NP) to 4-aminophenol (4-AP) by NaBH_4_ at room temperature (Figure 4a). The results were compared with Au NSphs with the same equivalent diameter and larger Au NSs of 20 nm. To monitor the chemical process, UV–vis extinction spectra were recorded by using the extinction band at 400 nm, which corresponds to the presence of 4-nitrophenolate (4-NP^–^), formed in alkaline media soon after injecting NaBH_4_. The solution instantly turned from pale yellow to bright yellow after adding NaBH_4_. After the catalyst (USNSs, NSs, or NSphs) was added, the chemical reduction started, with a color change from bright yellow to colorless and the concomitant gradual decrease of the 400 nm band and the appearance of a band at 300 nm. The reaction was initiated after an induction time (*t*_0_), which is the time related to consuming the dissolved oxygen by oxidizing 4-nitrosophenol and reoxidizing the reduced catalyst surface until sufficient depletion of dissolved oxygen.^45^ The catalytic performance of USNSs, NSs, and NSphs reveals a high catalytic conversion of 99.9%, but as seen in Figure 4a, there is a much faster reduction using USNSs than in the case of 20 nm NSs or equivalent NSphs. Given the high excess of NaBH_4_ with respect to 4-NP, it was appropriate to assume a constant concentration and a pseudo-first-order reaction kinetics (eq S3).^46,47^ The reaction catalyzed by USNSs showed a much higher rate constant (*k*) and shorter induction time (*k* = 0.04745 s^–1^, *t*_0_ = 25 s) than NSs (*k* = 0.02986 s^–1^, *t*_0_ = 40 s) and NSphs (*k* = 0.01875 s^–1^, *t*_0_ = 54 s) as shown in the kinetic profile (Figure 4b). This expected difference in reaction rates could be easily attributed to the larger surface-to-volume ratio compared to NSph and larger NSs and the presence of higher index and more reactive facets of USNSs compared to NSphs.

Comparison with some previously reported cases in the literature must be taken cautiously, as it depends on the reaction conditions such as concentrations of catalyst, 4-NP and NaBH_4_, and temperature (Table S8). Despite that, for the mild conditions used here, the present USNSs show a better catalytic activity than NSphs (*k* = 0.0107 s^–1^) for the reduction of 4-NP at room temperature.^48^ On the other hand, when compared with previously reported Au NSs, the USNSs show in general a high catalytic activity, above the average for the reduction of 4-NP.

### 3.2 Surface-Enhanced Raman Scattering

SERS performance of the synthesized USNSs was evaluated using 4-MBA as a model molecule. Measurements were performed in a colloidal dispersion at a fixed USNS concentration (0.2 mM Au atoms) and varying concentrations of 4-MBA. Figure 4c shows the spectra with the expected increase in signal intensity with an increasing concentration of the analyte. There are two SERS distinct and highly intense peaks for 4-MBA, one located at 1080 cm^–1^, assigned to aromatic ring breathing, symmetric C–H in-plane bending and C–S stretching, and the other at 1587 cm^–1^, assigned to ring C–C stretch and asymmetric C–H in-plane bending.^49^ Depending on the substrate used for measurements, the characteristic peak with the highest intensity is one or the other.^49^ In our case, the characteristic peak with the highest intensity was the one at 1080 cm^–1^, and therefore, it was used for evaluation. Figure 4d represents the intensity of that peak at different concentrations of MBA, exhibiting a linear regime in the 0.1–10 μM range and a limit of detection lower than 10 nM. On the other side, for the case of NSphs, no signal was detected for the equivalent MBA concentrations (Figure S7), which is expected since NSs are unique structures for SERS, exhibiting some of the sharpest features, which act as high-intense intrinsic hot spots, while spheres lacking any sharp edges would require an assembly to generate extrinsic hot spots at the nanoparticle contact points.

To evaluate the SERS capabilities of the synthesized USNSs, the enhancement factor (EF) was calculated according to eq S4. A value of 2.91 × 10^6^ EF was achieved for an illumination laser of 830 nm. This value is among the best EFs found for NSs in a colloidal suspension.^26^ Note here that, although higher values have been found, around 10^9^–10^10^, these correspond to measurements involving extrinsic hotspots such as the one formed between the NSs and flat substrates.^50,51^ Finally, we also observed an increase in the EF of 2–3 times when increasing the size to 20 nm, which we attribute to the position of their plasmonic band closer to the excitation laser used here at 830 nm instead of the size of the nanostar. As has been seen for nanorods, the smaller size, maintaining the shape and plasmon band position, could improve the EF due to a decrease of the scattering component of the extinction.^52^

### 3.3 Photothermia

Au NSs are promising photothermal agents for temperature-triggered drug release as well as thermal ablation of tumors.^53−55^ Here, the photothermal properties of USNSs were characterized by temperature monitoring of a colloidal solution (0.2 mM) under three laser wavelengths at the first biological transparency window (675, 785, and 808 nm) and a power of 0.33 W/cm^2^, which is normally considered the biological safety limits for skin illumination.^56−58^ The three wavelengths were chosen to be around the plasmonic band of the USNSs, below and above for 675, and 785–808 nm, respectively (Figure 4e). As a control, NSphs with the same equivalent diameter were also measured. Temperature change profiles during the heating and cooling phases are shown in Figure 4f. The USNSs revealed outstanding photothermal capabilities, with the temperature progressively increasing during the 20 min of irradiation and a final temperature increase between 15 and 20 °C depending on the illumination wavelength. In contrast, the NSphs did not show more temperature increase than the water control due to its negligible extinction at the NIR region.

The specific absorption rate (SAR) and photothermal conversion efficiency (η) were extracted from the thermograms following the corrected-losses method previously developed.^59^ All USNS samples showed relatively high SAR values of 3.3, 3.7, and 3.1 kW/g at 675, 785, and 808 nm laser wavelengths, respectively, which correspond to 10, 11.2, and 9.4 KW/g per w/cm^2^ of light irradiance. Here, the broadband of the USNSs (Figure 4e) has the positive consequence that those values do not have a significant variation for the whole first transparency window. The photothermal conversion efficiency revealed also good values for the USNSs, being η = 0.51, 0.54, and 0.48, for 675, 785, and 808 nm laser wavelengths, respectively, in line with values previously found for other Au anisotropic nanoparticles.^60−62^ Equally to the SAR, there are no significant changes among them, the highest value being that of 785 nm, which explains also its highest SAR, even when its extinction coefficient was slightly lower. Note here that although USNSs show η and SAR values in line with other NSs but not better, their small size could still be beneficial in nanobiomedical photothermal applications through the improvement of the biokinetics and therefore their concentration at the therapeutic region of interest. Moreover, the broad plasmonic band of USNSs, covering the first biological window, makes the results relatively constant, adds flexibility for the choice of illumination source, and makes the results more reliable under possible wavelength shifts occurring by the variation of the refractive index for different tissues.

## 4 Conclusions

We have studied the seed-mediated-growth synthesis of Au NSs with Cu^2+^-induced spike-sharpening effect and their optimization to generate USNSs. Tuning reaction parameters (PVP concentration, injection time, Cu^2+^ concentration, and seed volume) allow for controlling both the size and the optical properties of the NSs; at the optimal reaction parameters, sub-10 nm USNSs with a volume as small as 421 nm^3^, and an equivalent diameter of 9.2 nm, while exhibiting a plasmonic band at the NIR. Moreover, at those small sizes, regulating the amount of added Cu^2+^ presents an effective and linear way to fine-tune the optical properties of the NSs without changing the NS size.

ICP, XRD, EDX, and HR-TEM analysis indicated that Cu^2+^ helps in the creation of defects to generate tips at earlier stages of the NS growth, but only a small amount of it is incorporated in the NS, appearing homogeneously distributed and not affecting the lattice characteristics of the crystalline Au of the NS. The NSs also showed some degree of reshaping, which can be hindered by the correct choice of stabilizing molecules.

The obtained USNS were evaluated for different applications of high interest, including catalysis, SERS sensing, and photothermia. The catalytic conversion of 4-NP to 4-AP was evaluated by using USNSs as a catalyst. USNSs showed much higher catalytic activity than the equivalent NSphs and larger NSs, attributed to their higher surface-to-volume ratio and the existence of high Miller index facets of the USNSs. SERS sensing showed promising results with >10^6^ EF in colloidal dispersion. Photothermal measurements revealed SAR values of 3.1–3.7 kW/g under biological safety limits and photothermal conversion efficiencies of 0.48–0.51 at several wavelengths in the first biological transparency window. The current study enhances our understanding of the reaction parameters of USNSs, offering important insights for widespread applications due to their remarkable optical, catalytic, and photothermal properties.

## Supporting Information Available

Chemicals, detailed experimental procedures, characterization data and additional figures and tables (PDF)

## Abbreviations

NSs: gold nanostars
NIR: near IR
USNSs: ultrasmall NSs
LSPR: localized surface plasmon resonance
HEPES: 4-(2-hydroxyethyl)-1-piperazineethanesulfonic acid
EPPS: 2-(2-hydroxyethyl)-1-piperazinepropanesulfonic acid
MOPS: 3-(*N*-morpholino)propanesulfonic acid
SERS: surface-enhanced Raman scattering
PTT: photothermal therapy
ROC: radius of curvature
MUA: 11-mercaptoundecanoic acid
MBA: 4-mercaptobenzoic acid
PEG-SH: poly(ethylene glycol) methyl ether thiol
NSphs: nanospheres
4-NP: 4-nitrophenol
4-AP: aminophenol
*t*_0_: induction time
SAR: specific absorption rate
LOD: limit of detection
