# Supplementary material for: Cu2+–Assisted Synthesis of Ultrasharp and Sub-10 nm Gold Nanostars. Applications in Catalysis, Sensing, and Photothermia
Source: ACS Appl Nano Mater. 2024 Aug 15;7(16):19416–26. doi: 10.1021/acsanm.4c03310 (PMC11348798; doi:10.1021/acsanm.4c03310)
Supplement: Supplementary file 1 — an4c03310_si_001.pdf [file an4c03310_si_001.pdf]

Supporting Information

Cu^{2+}-assisted Synthesis of Ultrasharp and Sub-10 nm Gold Nanostars. Applications in Catalysis, Sensing, and Photothermia

*Esraa Samy Abu Serea,[1] Leixuri B. Berganza,[1] Senentxu Lanceros-Méndez,[1,2] and Javier Reguera.[1,3]**

[1] BCMaterials, Basque Center for Materials, Applications, and Nanostructures, UPV/EHU Science Park, Leioa 48940, Spain.

[2] Ikerbasque, Basque Foundation for Science Bilbao, 48009 Bilbao, Spain.

[3] University of Valladolid, Dept. Condensed Matter Physics, Bioforge, Pso. de Belén 19 47011, Valladolid, Spain.

Email: javier.reguera@uva.es

Contents

Experimental section 2
- Chemicals 2
- Synthesis 2
  - Synthesis of 3.7 nm spherical Au seeds 2
  - The pre-reduction kinetics 3
  - Tuning injection time of Au seeds 3
  - Tuning the concentration of PVP 4
  - Tuning volume of $CuCl_2 \cdot 2H_2O$ 4
  - Tuning volume of PVP-coated Au seeds 4
  - Synthesis of gold USNSs for structural characterization and applications 4
  - Synthesis of corresponding gold NSphs 5
  - Synthesis of 20 nm NSs 5
  - Purification process 6
  - Surface functionalization of USNSs (reshaping experiment) 6

Supporting tables 6
- Characterization 11
  - Determination of the smallest NS size 11
  - Chemical reduction in the formation of USNSs 11
  - Electron Microscopy 12
  - UV−Vis Spectroscopy 13
  - ICP-AES Elemental Analysis 13
  - X-ray Diffraction 13

Applications experimental conditions 14
- Catalytic reduction of 4-nitrophenol 14
- Surface Enhanced Raman Scattering (SERS) 14
- Photothermal Measurements 16

Supporting figures 16

Supporting references 19

Experimental section

Chemicals

Hydrogen tetrachloroaurate trihydrate (99.99%, Alfa); N,N-dimethylformamide (99.9%, labKem), Sodium borohydride (98%, Alfa); Polyvinylpyrrolidone (PVP) (MW= 10 kg mol^{-1}, Tokyo chemical industry (TCI)); Sodium citrate dihydrate (≥99.0%, Sigma-Aldrich); Tannic acid (≥99.0%, Sigma-Aldrich); Potassium carbonate ACS reagent (≥99.0%, Sigma-Aldrich); 4-nitrophenol PESTANAL® (Sigma-Aldrich); Copper(II) chloride dehydrate ACS reagent (≥99.0%, Sigma-Aldrich); 4-Mercaptobenzoic acid (99%, Sigma-Aldrich); 11-Mercaptoundecanoic acid (95%, Sigma-Aldrich); Poly(ethylene glycol) methyl ether thiol (average M_n 2,000 and 6000, Sigma-Aldrich); absolute ethanol (≥99.9%). The reagents were used as received without any further purification.

Synthesis

Synthesis of 3.7 nm spherical Au seeds

Citrate-coated spherical Au seeds were synthesized as previously reported. [1] The synthesis started by injecting 1 mL of $HAuCl_4 \cdot 3H_2O$ (25 mM) into a freshly prepared solution mixture containing 150 mL of sodium citrate (2.2 mM), 0.1 mL of (2.5 mM) tannic acid, and 1 mL of potassium carbonate (150 mM) in a 250 mL necked round flask at 70 ºC. The initially transparent solution turned to an orange-red colour. The solution was maintained at this temperature for 10 minutes to ensure the full conversion of the gold precursor. The solution was left to cool down, measured by UV-Vis to check the quality of the seeds, and stored in the fridge until further use. The final Au seeds showed a diameter of 3.7 ± 0.46 nm and a LSPR band at 510 nm (**Figure S2**).

The formed gold seed particles were then coated with PVP using approximately 60 molecules per nm^2 of Au surface. In this case, 5 mL of PVP solution (5.7 mM) was added drop-wise under

stirring to 100 mL Au seeds for 30 min to ensure the complete adsorption of PVP on Au seeds. The solution was then purified and concentrated by using a 15 mL Ultra-Filtration Centrifugal Tube (MWCO 30000) at 6000 g for 15 min. The filtrate was collected using 4 mL of Milli-Q water to obtain a final Au concentration of 4.83 mM. This concentration was calculated based on UV-Vis measurements in a 1 cm depth cuvette and assuming a 1.2 absorbance at 400 nm for 0.5 mM Au concentration.

The pre-reduction kinetics

Freshly dissolved solution of PVP (M_W: 10k) (mass quantities in **Table S2**) in 1 mL DMF was transferred to a quartz cuvette, then 5.46 μL of $HAuCl_4 \cdot 3H_2O$ (50 mM) was injected (**Table S.2** shows the used quantities). Monitoring the reduction rate of Au precursor from Au^{3+} to Au^{1+} was performed by UV-Vis spectroscopy using the Au^{3+} 330 nm band. For much longer times (not shown here) an increase in the absorbance at this wavelength is also observed due to the $Au^{1+} \rightarrow Au^0$ and the appearance of a broad band in the 600-700 nm from the appearance of self-nucleated NSs.

Tuning injection time of Au seeds

$HAuCl_4 \cdot 3H_2O$ (5.46 μL, 50 mM) was added to DMF solution of 200 mg/mL of PVP (M_W: 10k). The mixture was stirred and monitored by UV-Vis following the description of the previous section. The time for a given reduction in absorbance was calculated. The times for the given relative concentrations of Au^{3+} was: (7%) 47 s, (6%) 48 s, (6%) 51 s, (4%) 57 s, (3%) 63 s, (1%) 83 s (**Table S3**).

The syntheses of Au NSs were performed by rapidly adding the PVP-coated Au seeds (1.6 μL, 4.83 mM) together with $CuCl_2 \cdot 2H_2O$ (38.53 μL, 5 mM) at the defined injection times. Soon

after, the solution turned blue and was left stirring for 20 min, centrifuged, re-suspended in ultrapure water once, and then measured by UV-Vis.

Tuning the concentration of PVP

The same synthetic procedure as the previously above described, but varying the PVP concentration according to **Table S3** was performed here. The injection time was selected as the 96 % prereduction (4% of remaining Au^{3+}) for all cases **(Table S4).**

Tuning volume of $CuCl_2 \cdot 2H_2O$

$HAuCl_4 \cdot 3H_2O$ (5.46 µL, 50 mM) was added to a freshly prepared DMF solution of 200 mg/mL PVP at room temperature. The mixture was stirred at a pre-reduction time of 57 s, and then PVP-coated Au seeds (1.6 µl, 4.83 mM) and a varying volume of $CuCl_2 \cdot 2H_2O$ (5, 15, 20, 25, 30, 35, 40, 60, 100, 150 µL, 5 mM) **(Table S5)** were simultaneously and quickly added. The solution was left stirred for 20 min. The solution was centrifuged once, re-dispersed in water, and measured by UV-Vis.

Tuning volume of PVP-coated Au seeds

The same synthetic procedure, as previously described for the case of changing the injection time, was used here for the synthesis of NSs with and without Cu^{2+} addition. For the NSs with Cu^{2+} addition, the only modification was the volume of PVP-coated Au seeds (**Table S6**), which was (50, 30, 20, 15, 10, 5, 3, 2, 1 µL, 1 Mm). The same volume variations of the seeds were used for the synthesis of normal NSs without Cu^{2+} addition as provided in **Table S7.**

Synthesis of gold USNSs for structural characterization and applications

$HAuCl_4 \cdot 3H_2O$ (5.46 µl, 50 mM) was added to a solution containing 200 mg of PVP, completely dissolved in 1 ml DMF, and then stirred for 57 s. Soon after, PVP-coated Au seeds (15 µL, 1 Mm) and $CuCl_2 \cdot 2H_2O$ (40 µL, 5 mM) were rapidly added. The solution was left stirring for 20 min at room temperature.

The synthesis of these USNSs was critically dependant on the PVP used in the reaction. For instance, when a Sigma-Aldrich brand was used (MW:10 Kg/mol, batch: WXBC8072V), the smallest achievable size with absorbance at 700 nm was14.6 nm, while we have observed batches that did not shown any reductant capability (Sigma-Aldrich, MW:10 Kg/mol, batch: WXBD7573V).

Synthesis of corresponding gold NSphs

Au nanospheres (NSphs) with the same volume as the above USNSs were obtained as previously reported.[2] The synthesis reagent quantities used for USNSs remained constant with only two variations: (i) $CuCl_2 \cdot 2H_2O$ was not added, and (ii) immediately after the injection of Au seeds the solution temperature was increased to 120 °C by inserting the reaction flask into an oil bath. The reaction was left stirring at that temperature for at least 20 minutes, followed by cooling the flask down to room temperature. At the end of the reaction, the solution became red, indicative of the formation of quasi-spherical nanoparticles. The solution was centrifuged and measured by UV-Vis.

Synthesis of 20 nm NSs

$HAuCl_4 \cdot 3H_2O$ (5.46 µL, 50 mM) was added to a freshly prepared DMF solution of 200 mg/mL PVP at room temperature. The mixture was stirred at a pre-reduction time of 57 s, and then PVP-coated Au seeds (0.6 µl, 4.83 mM). The solution was left stirred for 20 min.

Purification process

Directly after the synthesis, the nanoparticle solution was washed with ethanol by centrifugation 3-4 times (15×10^3 g, 30 min). Afterward, the precipitate was redispersed in 1 mL Milli-Q water and refrigerated.

Surface functionalization of USNSs (reshaping experiment)

0.5 mL (70 μM) of different ligands solutions: MUA, PEG-SH (M_n 2,000), MBA, and PEG-SH (M_n 6,000), were added dropwise to 4 vails with equal volume of previously prepared USNSs (0.5 mL, 0.1 mM), respectively. The reaction was left stirring for 1 hour at room temperature. To have approximately 100 monomer units for each nm^2 nanoparticle surface, the ligand volumes were determined based on the concentration of nanoparticle. Subsequently, all sample solutions were washed by centrifugation once (15×10^3 g, 30 min), and the supernatant (with the non-bound ligands) was discarded. The precipitate was then redispersed in 0.5 mL Milli-Q water. Following functionalization, kinetic measurements were performed by tracking the UV-Vis spectra to functionalized USNSs during the period of incubation at room temperature for several months.

Supporting tables

Table S1: Examples of Au NSs reported in the literature. Range of sizes obtained, their synthesis technique, and LSPR peak positions

Shape/profile	Synthesis method	Reducing agent	Size	LSPR peaks	Sharpness (nm)	Ref.
AuNSs	seed-mediated, silver-assisted	CTAB	200−400	550−820	2.365 ± 0.317	[3]
AuNSs	seedless, silver-assisted	Triton-X-100	100−300	600−1100	3.794 ± 1.062	[4]
Pentagonal AuCuNSs	seed-mediated	Oleylamine (OLA) hexadecylamine (HDA)	130−220	520−1050	13.250 ± 0.649	[5]

AuNSs	seed-mediated	Ascorbic acid	47−214	718−1015	-	6
AuNSs	seed-mediated, silver-assisted	Triton-X-100	100−160	550−1000	2.388 ± 0.486	7
AuNSs	Seedless	Good's buffers	50−150	650−950	3.161 ± 0.131	8
AuNSs	seed-mediated	PVP	45−116	700−850	3.598 ± 0.741	9
AuNSs	seed-mediated	Ascorbic acid	105	611	-	10
AuNSs	seed-mediated	Good's buffers	96	500−950	3.236 ± 0.674	11
AuNSs	seed-mediated	Ascorbic acid	86	750	-	12
AuNSs	seed-mediated	Ascorbic acid	80	700	3.291 ± 0.365	13
AuNSs	Seedless	Ascorbic acid	75	630	2.09 ± 0.248	14
AuNS@Ag	seed-mediated, silver coating	CTAB	50−70	500−850	2.322 ± 0.349	15
AuNSs	seed-mediated	HEPES	40−60	708−720	-	16
AuNSs	seed-mediated	PVP	60	767	3.415 ± 0.668	17
AuNSs	seed-mediated, silver-assisted	ascorbic acid	50	750	2.212 ± 0.231	18
AuNSs	seed-mediated, surfactant-free	ascorbic acid	50	800	2.355 ± 0.242	19
AuNSs	seed-mediated	PVP	41	785	2.482 ± 0.471	20
AuNSs	seed-mediated, surfactant-free	ascorbic acid	40	800	3.203 ± 0.572	21
AuNSs	seedless	EPPS	43	700	4.065 ± 0.961	22
AuNSs	seed-mediated	PVP	35	750	6.507 ± 1.094	23
AuNSs	Seedless	HEPES	35	580	2.373 ± 0.539	24
AuNSs	seed-mediated	CTAB	20-30	650	3.361 ± 0.469	25
AuNSs	seed-mediated	PVP	22−30	700−750	3.009 ± 0.441	26
AuNSs	seedless	HEPES	29	700	3.480 ± 0.435	27
AuNSs	seed-mediated, silver-assisted	ascorbic acid CTAB	100	780	6.566 ± 1.684	28

Table S2: Pre-reduction kinetics of Au^{3+}.

**$Weight_{PVP}$ [mg]**	**V_{DMF} [mL]**	**$V_{HAuCl_4.3H_2O}$ 50mM [µL]**	**Injection Time 4% [s]**
50	1	5.46	300
100	1	5.46	120
125	1	5.46	97
140	1	5.46	95
150	1	5.46	73
175	1	5.46	75

200	1	5.46	57
225	1	5.46	47
250	1	5.46	44
300	1	5.46	40
350	1	5.46	42

Table S3: Tuning injection time of Au seeds.

Weight$_{PVP}$ [mg]	V_{DMF} [mL]	$V_{HAuCl_4.3H_2O}$ (50 mM) [µL]	Injection Time [s]	V_{Seeds} (4.83 mM) [µL]	V_{CuCl_2} (5 mM) [µL]
200	1	5.46	(7%) 47	1.6	38.53
200	1	5.46	(6%) 48	1.6	38.53
200	1	5.46	(5%) 51	1.6	38.53
200	1	5.46	(4%) 57	1.6	38.53
200	1	5.46	(3%) 63	1.6	38.53
200	1	5.46	(2%) 70	1.6	38.53
200	1	5.46	(1%) 83	1.6	38.53

Table S4: Tuning the concentration of PVP.

Weight$_{PVP}$ [mg]	V_{DMF} [mL]	$V_{HAuCl_4.3H_2O}$ (50 mM) [µL]	Injection Time 4% [s]	V_{Seeds} (4.83 mM) [µL]	V_{CuCl_2} (5 mM) [µL]
50	1	5.46	300	1.6	38.53
100	1	5.46	120	1.6	38.53
125	1	5.46	97	1.6	38.53
140	1	5.46	95	1.6	38.53
150	1	5.46	73	1.6	38.53
175	1	5.46	75	1.6	38.53
200	1	5.46	57	1.6	38.53
225	1	5.46	47	1.6	38.53
250	1	5.46	44	1.6	38.53
300	1	5.46	40	1.6	38.53
350	1	5.46	42	1.6	38.53

Table S5: Tuning volume of $CuCl_2 \cdot 2H_2O$.

Weight$_{PVP}$ [mg]	V_{DMF} [mL]	$V_{HAuCl_4.3H_2O}$ (50 mM) [µL]	Injection Time 4% [s]	V_{Seeds} (4.83 mM) [µL]	V_{CuCl_2} (5 mM) [µL]

200	1	5.46	57	1.6	5
200	1	5.46	57	1.6	15
200	1	5.46	57	1.6	20
200	1	5.46	57	1.6	25
200	1	5.46	57	1.6	30
200	1	5.46	57	1.6	35
200	1	5.46	57	1.6	40
200	1	5.46	57	1.6	60
200	1	5.46	57	1.6	100
200	1	5.46	57	1.6	150

Table S6: Tuning volume of PVP-coated Au seeds for NSs synthesis with Cu^{2+} addition.

**Weight$_{PVP}$ [mg]**	**V_{DMF} [mL]**	**$V_{HAuCl_4.3H_2O}$ (50 mM) [µL]**	**Injection Time 4% [s]**	**V_{Seeds} (1 mM) [µL]**	**V_{CuCl_2} (5 mM) [µL]**	**$(V_{Au3+}/V_{seeds})^{1/3}$**
200	1	5.46	57	50	40	0.477977602
200	1	5.46	57	30	40	0.566705111
200	1	5.46	57	20	40	0.648715412
200	1	5.46	57	15	40	0.714003698
200	1	5.46	57	10	40	0.817330203
200	1	5.46	57	5	40	1.029771527
200	1	5.46	57	3	40	1.22092915
200	1	5.46	57	2	40	1.397614987
200	1	5.46	57	1	40	1.760884542

Table S7: Tuning volume of PVP-coated Au seeds for NSs synthesis without Cu^{2+} addition.

**Weight$_{PVP}$ [mg]**	**V_{DMF} [mL]**	**$V_{HAuCl_4.3H_2O}$ (50 mM) [µL]**	**Injection Time 4% [s]**	**V_{Seeds} (1 mM) [µL]**	**$(V_{Au^{3+}}/V_{seeds})^{1/3}$**
200	1	5.46	57	50	0.477977602
200	1	5.46	57	30	0.566705111
200	1	5.46	57	20	0.648715412
200	1	5.46	57	15	0.714003698
200	1	5.46	57	10	0.817330203
200	1	5.46	57	5	1.029771527
200	1	5.46	57	3	1.22092915
200	1	5.46	57	2	1.397614987
200	1	5.46	57	1	1.760884542

Table S8: Comparison of the catalyst performance in the reduction of 4-nitrophenol (4-NP) using different types of gold nanoparticles from the literature at room temperature.

Catalyst	Size (nm)	Rate constant at room temperature (min^{-})	Reaction time (min)	$NaBH_4$/4-NP/Au (molar ratio)	Ref.
AuNSphs	15	0.25×10^{-3}	3	51.21/0.027/1	[29]
AuNSphs	26	0.24×10^{-3}	3	51.21/0.027/1	[29]
AuNSphs	34	0.18×10^{-3}	5	51.21/0.027/1	[29]
AuNSphs	4	1.25	–	516/1.72/1	[30]
AuNSphs	16	0.36	–	516/1.72/1	[30]
AuNSphs	40	0.23	–	516/1.72/1	[30]
AuNSs	38	1.78	–	516/1.72/1	[30]
AuNSs	117	4.36	–	516/1.72/1	[30]
AuNSs (8-10 tips)	–	0.22	17	15.932/23.28/1	[31]
AuNSs (6-8 tips)	–	0.18	25	15.932/23.28/1	[31]
AuNSs (4-6 tips)	–	0.12	35	15.932/23.28/1	[31]
AuNFs (nanoflower)	52	0.039×10^{-3}	–	153.37/76.69/1	[32]
AuNSphs	30-50	0.025×10^{-3}	–	153.37/76.69/1	[32]
AuNSs	60–70	0.21	13	19.69/0.30/1	[33]
AuNFs	100	0.13	29	19.69/0.30/1	[33]
Au nanosnowflakes	90	0.44	7	19.69/0.30/1	[33]
AuNSs	31	6.37×10^{-2}	45	–	[34]
AuNSphs	40	6.75×10^{-3}	112	–	[34]
AuNSphs	28	1.53×10^{-2}	64	–	[34]
AuNSphs	12	2.85×10^{-2}	64	–	[34]
AuNSphs	10-12	0.745	5	7813/78.13/1	[35]

Table S9. Wavenumbers and band assignments of SERS band of 4-MBA. The peak at 880 cm^{-1}, which remains constant despite the change in the concentration of the analyte, corresponds to USNSs.

Wavenumber (cm^{-1})	Band assignment	Reference
1587	C–C stretching and asymmetric C–H in-plane bending	[36,37]
1485	Ring bending	[37]
1272		

1179	C-H deformation	[37,38]
1080	Aromatic ring breathing, symmetric C–H in-plane bending and C–S stretching	[36,38]
800	COO^- stretching	[37,38]
694		
521	Ring out-of-plane bending	[37]
335		

Characterization

Determination of the smallest NS size

Figure 2i shows the maximum wavelength (λ_{max}) as a function of their seed-to-growth volume ratio and the equivalent diameter. The diameter was calculated by synthesizing spherical nanoparticles with the same volume and using a calibration line of size versus volume ratios **(Figure S3)**. The smallest size of NSs (defined as the smallest size with a λ_{max} higher than 700 nm), with and without Cu, were obtained by fitting the empirical sigmoidal function, **Equation 1**: [39]

$$\lambda_{max} = A_2 + \frac{(A_1 - A_2)}{1 + e^{\frac{(d - d_0)}{dx}}} \quad (S1)$$

where A_1, A_2, dx and d_0 are fitting constants. A_1 and A_2 correspond to the minimum and maximum λ_{max}, d_0 is the center value, and dx indicates how rapidly the variation takes place. The smallest size is obtained for λ_{max} = 700 nm and subtracting d from **Equation 1**.

Chemical reduction in the formation of USNSs

During the synthesis of USNSs, PVP is oxidized and reduces the Au^{3+} of the salt $HAuCl_4 \cdot 3H_2O$ first to Au^{1+} (pre-reduction step) and then, once the gold seeds are added to metallic gold (Au^0). The Cu^{2+} added together with the seeds is only partially reduced to metallic Cu (Cu^0) as shown in the ICP results. This agrees with the standard reduction potential (half-reaction) in the production of those elements as reported in the literature: [40–42]

$AuCl_4^- + 2e^- \rightarrow AuCl_2^- + 2Cl^-$ $E^0 = 0.926$ V

$AuCl_2^- + e^- \rightarrow Au^0 + 2Cl^-$ $E^0 = 1.154$ V

$Cu^{2+} + 2e^- \rightarrow Cu^0$ $E^0 = 0.34$ V

Electron Microscopy

Transmission electron microscopy (TEM) samples were prepared by placing a 2 μL drop of NP solution on carbon-coated Cu 400-square mesh grids and allowing it to dry at room temperature carbon-coated Ni grids were used for the case of EDX mapping. All samples were imaged using JEM-1400PLUS, JEOL equipment operated at 100 kV. High-resolution TEM (HR-TEM) images were captured using JEOL (JEM-FS2200 HRP) instrument working at 200 KV. HAADF-STEM, and EDX-STEM measurements were performed in a Thermo Fisher Talos- F200i, working at 200 kV. Selected-area EDX spectrum was performed on a QUANTA 200 FEG ESEM equipped with an EDAX Genesis. In this case, a drop with the nanoparticle solution was dropped on a carbon double tape on the aluminium support and left to dry.

ImageJ software program package (FIJI) was used to analyse the images. The equivalent diameter was measured on the NSphs from the projected area and assuming completely spherical particles. Those diameters were used to obtain a calibration that allowed to know the volume of equivalent NSs **(Figure S3)**. To obtain the radius of curvature, the circle selection tool was used to fit the contour at the tip of the spikes, and the radius was taken from the area of that selection. For the analysis of images of previously reported articles, the images were re-scaled and filtered using the filter median (2px) to avoid pixelation problems during the selection.

UV−Vis Spectroscopy

Optical characterization of aqueous solutions of USNSs and NSphs was typically performed in a glass cuvette using UV−vis spectroscopy (Cary60, Agilent) in the range from 350 nm to 1100 nm. UV−vis spectroscopy (Avantes: Avaspec ULS spectrometer and AvaLight-DHc as a light source) was used in the range 200−500 nm for catalysis measurements in quartz cuvettes by placing the sample holder on top of a magnetic stirring plate.

ICP-AES Elemental Analysis

Elemental analysis was studied using inductively coupled plasma (Agilent 5100) atomic emission spectroscopy (ICP-AES), using a quartz spray concentric nebulizer and a Scott-type spray chamber. 100 µL of USNSs were digested with 200 µL aqua regia (150 µL HCl, and 50 µL HNO_3, trace analysis grade, added sequentially) and left for 1h. The sample was then diluted and mixed with 9.7 mL milli-Q water (final volume: 10 mL) before measurement by ICP-AES.

X-ray Diffraction

X-ray Diffraction (XRD) analyses were conducted using a PHILIPS X'PERT PRO automatic diffractometer. Operating conditions were at 40 kV and 40 mA, in a theta–theta configuration a secondary monochromator with Cu-Kα radiation (λ = 1.5418 Å), and a PIXcel solid-state detector (active length in 2θ 3.347°). The samples were placed on an Eulerian cradle with an X-Y-Z stage that was automatically controlled. **Figure S4** shows the diffractograms for USNSs and NSs, with the peaks fitting well to metallic Au.

Crystallite size was calculated by the Scherrer equation (**Equation 2**).[43]

$$\tau = \frac{K\lambda}{\beta\cos\theta} \quad \text{(S2)}$$

where τ is the mean size of crystal domains, K is a dimensionless shape factor (taken as 0.9 here), λ the X-ray wavelength, β the line broadening at half the maximum intensity (FWHM) and θ the Bragg angle, after subtracting the equipment broadening. Values corresponding to

the (111) peak were used for the calculation of crystallite size. For the lattice parameter calculation, the peak position with the highest intensity (111) was used.

Applications experimental conditions

Catalytic reduction of 4-nitrophenol

In a typical catalytic reaction, 4-nitrophenol (75 μL, 1.4 mM) was mixed together with $NaBH_4$ (78 μL, 0.4 M, freshly prepared in a water-ice bath). The solution instantly changed from pale to bright yellow. Soon after, a colloidal suspension of Au nanoparticles (USNSs, NSs or NSphs) (1.347 mL, 0.05 mM) was quickly added. The 4-NP catalytic reduction was monitored until the reaction was complete using a 1 cm path quartz cuvette. Spectra were taken every 1 s by UV-Vis spectroscopy in the 250–550 nm range at room temperature (20-21 ºC). The absorbance at 400 nm was represented as a function of time, giving rise to two regions, an initial time where no change was observed, followed by an exponential decay. This second region was fitted using a 1st-order kinetic model. The kinetic constant (k) was estimated according to the following equation (**Equation 3**):

$$\ln\frac{C}{C_0} = -\mathrm{kt} \quad (\mathrm{S3})$$

Where C_0 is the initial concentration, and C is the concentration at a given time t.

Surface Enhanced Raman Scattering (SERS)

SERS measurements were performed using a Raman instrument (Raman inVia Qontor Spectrometer) with an 830 nm excitation laser. The measurements were performed in a colloidal solution, fixing the USNSs content (0.2 mM Au) and varying MBA concentration. For that, 50 μL USNSs and 50 μL MBA of different concentrations were mixed, preparing solutions with a final concentration of 0.2 mM Au and a range of MBA concentrations (100,

10, 5, 1, 0.1, 0.01 μM). These solutions were shacked mechanically for 30 min before measurements. All SERS spectra were collected using a 50-objective magnification (numerical aperture NA = 0.65) with an integration time of 20 s and a laser power at 10% (~15mW). All spectra were treated by correcting the background baseline with the equipment software and smoothing and analysis using Origin 8.5 software.

The limit of detection (LOD) was taken as the minimum MBA concentration detected (by varying this one order of magnitude at a time). The enhancement factor (EF) was calculated according to the following formula (**Equation 4**).[44,45]

$$EF = \frac{\frac{I_{SERS}}{N_{SERS}}}{\frac{I_{Raman}}{N_{Raman}}} \quad (S4)$$

where I_{SERS} and I_{Raman} are the intensity, or count number, received by the instrument under the same irradiation conditions (power, wavelength and time), for SERS and Raman, respectively. This intensity was weighed by the laser power used and time, which can be different for the Raman and SERS measurements. For the case of SERS, the sample was measured in colloidal solution, and in Raman, given the much lower signal, it was measured as powder. N_{SERS} and N_{Raman} are the number of molecules measured for SERS and Raman, respectively. The number of molecules in SERS is calculated by multiplying the concentration of 4-MBA in solution, C, by the Avogadro's number and with laser focus effective volume (V_f). V_f is obtained by (laser spot diameter)$^2 \cdot \pi^{3/2}$, where laser spot diameter is obtained by ($1.22 \cdot \lambda/NA$),[46] where λ is the wavelength and NA the numerical aperture of the laser. For the case of Raman, the N_{Raman} was calculated based on the 4-MBA density, multiplying this vale by V_f, and the Avogadro's number and dividing by the 4-MBA molecular weight.

Photothermal Measurements

A reflector collimator (Thorlabs) coupled with a (675, 785, and 808 nm) laser (Lumics, LuOceanMini4) was used to have a collimated beam of about 12 mm in diameter. The light was transmitted through a 1×1 cm^2 mask located in front of a standard glass cuvette with a 1 cm^2 section. This cuvette held 1 mL of the nanoparticle solution at an Au concentration of 0.2 mM. To measure the light power, both before and after the sample was inserted, a thermal power meter (S425 Thorlabs) was also located behind the sample. To induce the photothermal effect of the nanoparticles, an illumination power of 0.33 W cm^{-2} was applied. A series of on/off experiments, with durations of 20 and 30 minutes respectively, were performed. The temperature was recorded using the Flir tools + program and an infrared thermal imaging camera (Flir E4) that was located on top of the cuvette. The specific absorption rate (SAR) and the photothermal conversion efficiency (η) were obtained from those thermograms using a corrected-losses method (full step-by-step method in the SI).[2]

Supporting figures

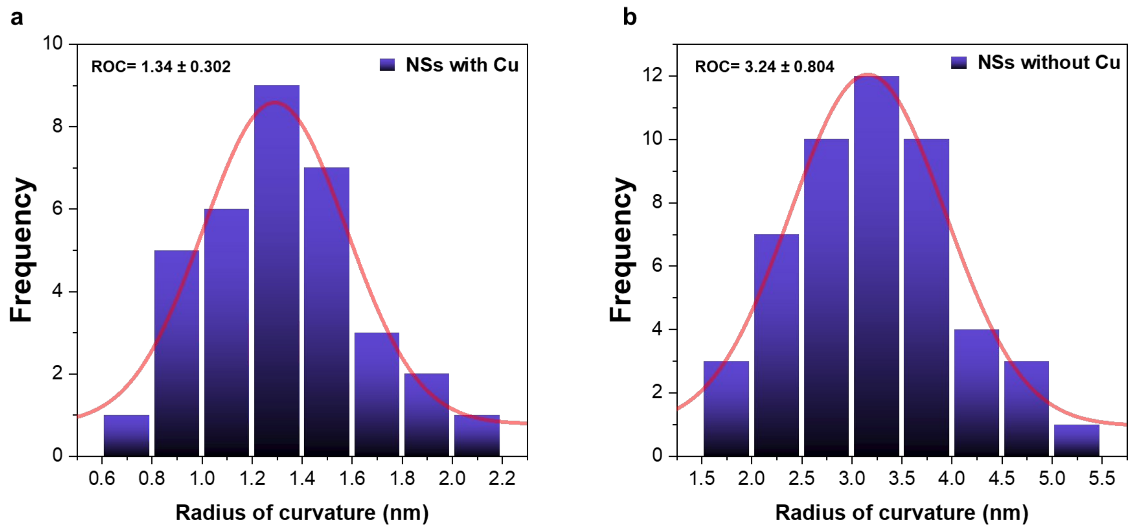

Figure S1. Statistical characterization of the radius of curvature (ROC) of the spike tips of (a) NSs with Cu^{2+} and (b) NSs without Cu^{2+}.

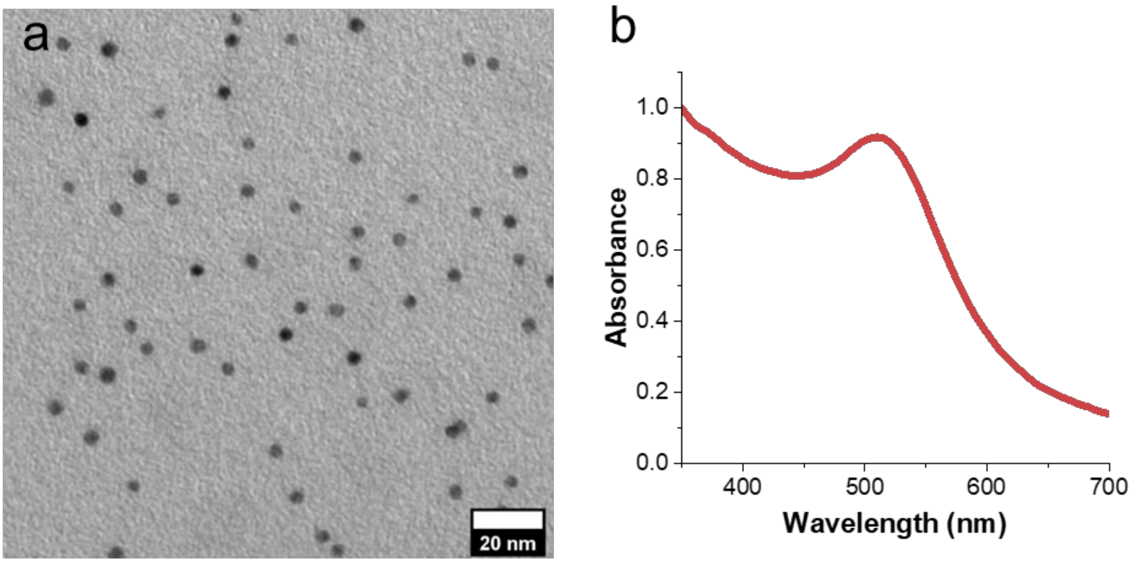

Figure S2. (a) TEM of Au seeds and (b) UV-Vis spectra of those seeds.

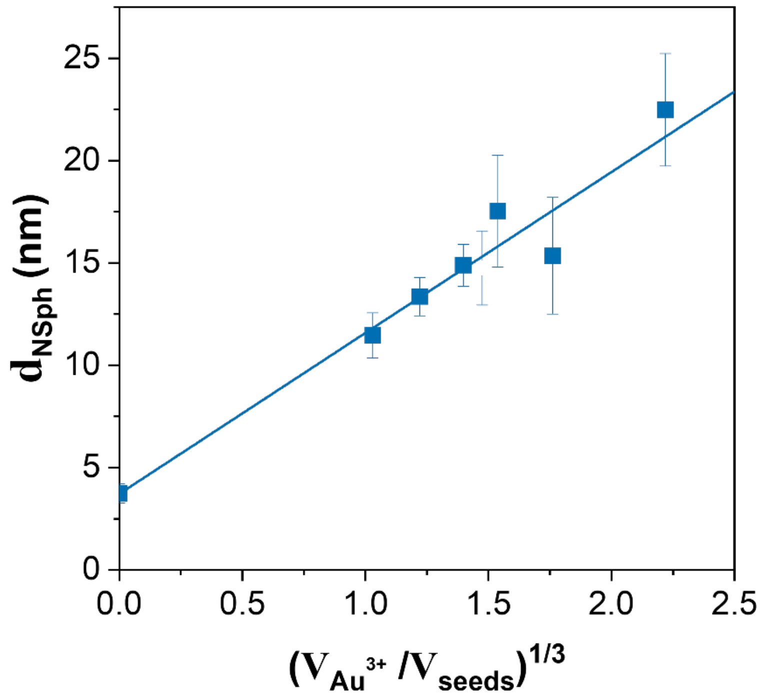

Figure S3. Calibration curve of volume ratio and diameter equivalent of the nanospheres.

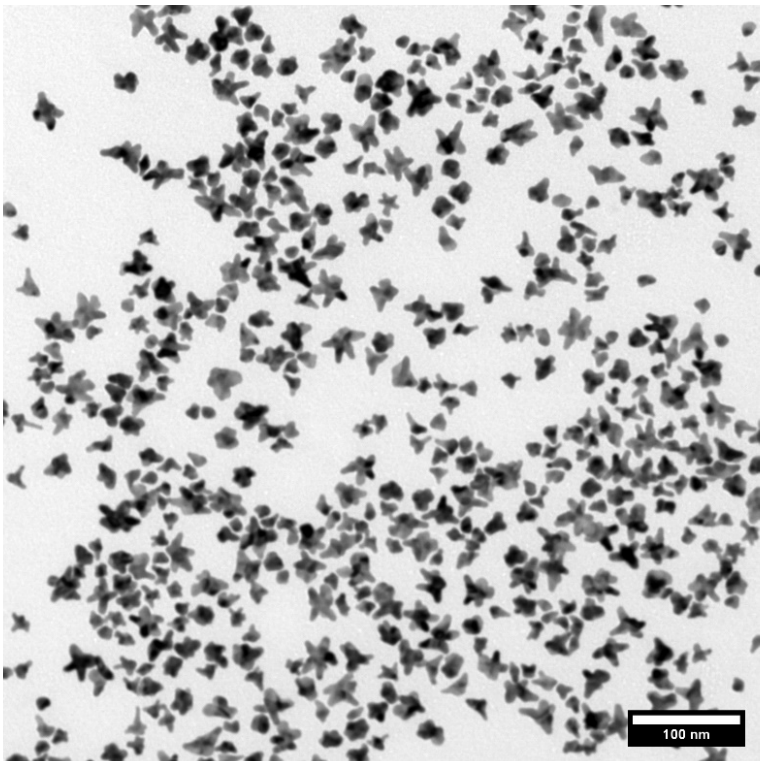

Figure S4. TEM image at low magnification of gold USNSs show relatively high yield of the synthesis method. The obtained USNSs have more than one spike on their surface.

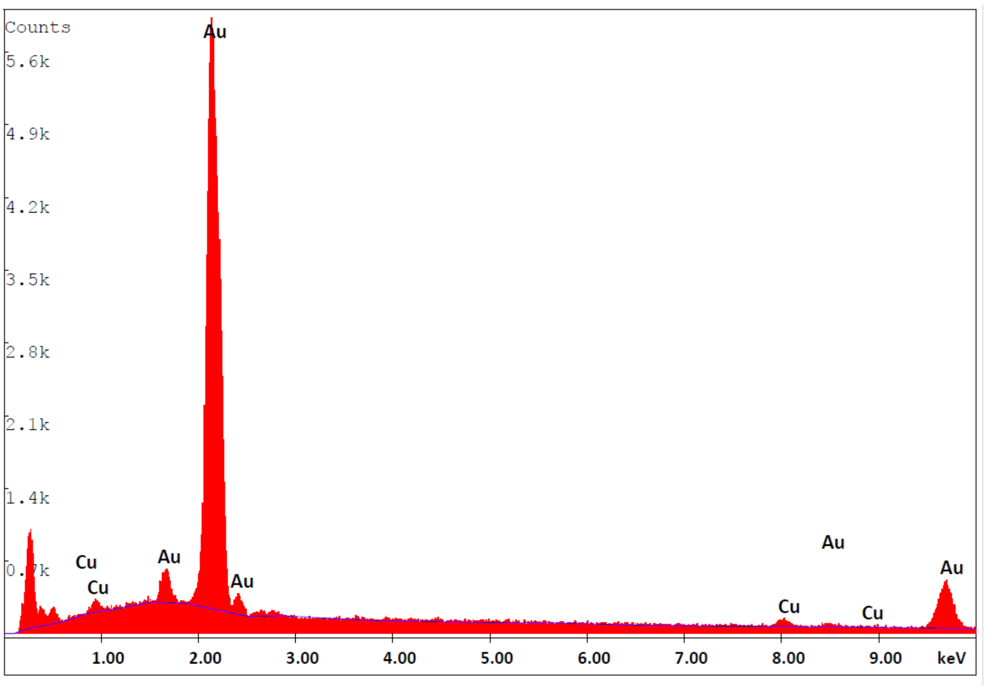

Figure S5. EDX spectrum of USNSs showing the main presence of Au and a much lower quantity of Cu. The semiquantitative analysis using Au-M and Cu-K energy bands shows a weight mass distribution of 98.05 and 1.95 % wt. for Au and Cu respectively.

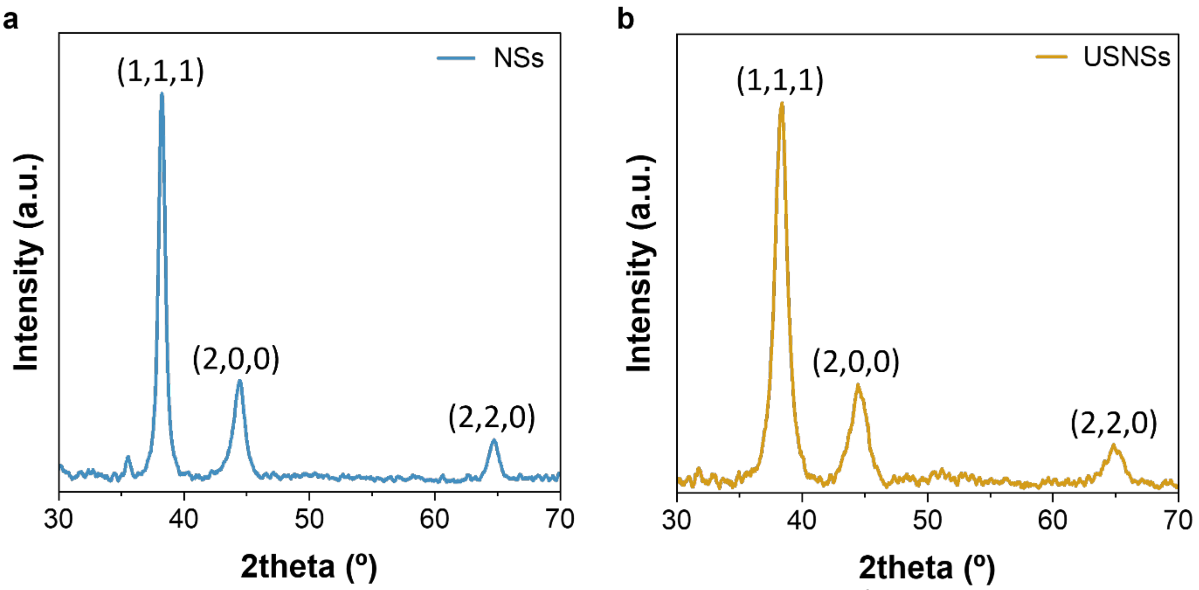

Figure S6. X-ray diffraction pattern of (a) standard NSs without Cu^{2+}, and (b) USNSs with Cu^{2+}.

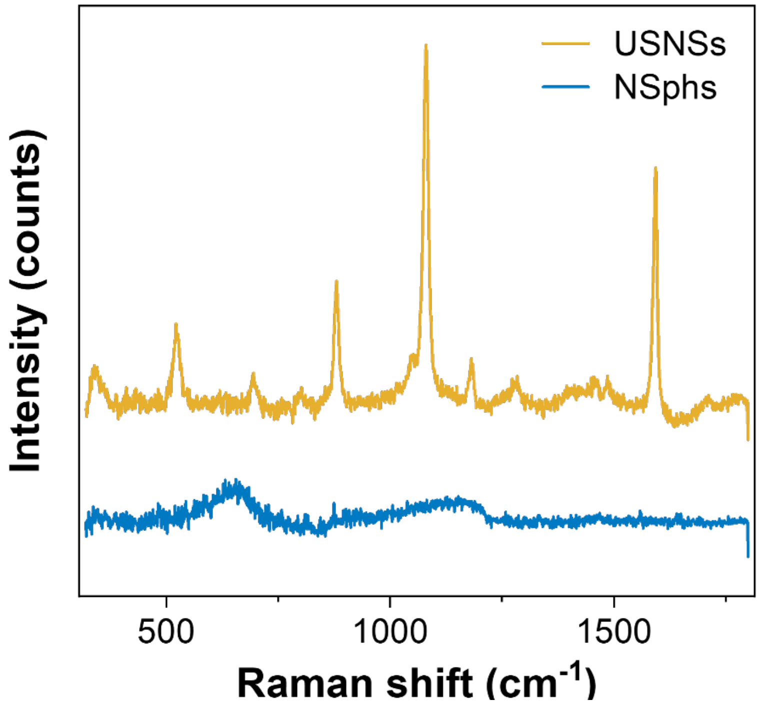

Figure S7. Comparative SERS spectra of 4- MBA (100 µM) using USNSs and NSs with the same equivalent diameter and at the same concentration.

Supporting references

(1) Piella, J.; Bastús, N. G.; Puntes, V. Size-Controlled Synthesis of Sub-10-Nanometer Citrate-Stabilized Gold Nanoparticles and Related Optical Properties. *Chem. Mater.* **2016**, *28* (4), 1066–1075. https://doi.org/10.1021/acs.chemmater.5b04406.

(2) Abu Serea, E. S.; Orue, I.; García, J. Á.; Lanceros-Méndez, S.; Reguera, J. Enhancement

and Tunability of Plasmonic-Magnetic Hyperthermia through Shape and Size Control of Au:Fe 3 O 4 Janus Nanoparticles. *ACS Appl. Nano Mater* **2023**, *6*, 18466–18479. https://doi.org/10.1021/acsanm.3c03818.

(3) Chatterjee, H.; Rahman, D. S.; Sengupta, M.; Ghosh, S. K. Gold Nanostars in Plasmonic Photothermal Therapy: The Role of Tip Heads in the Thermoplasmonic Landscape. *J. Phys. Chem. C* **2018**, *122* (24), 13082–13094. https://doi.org/10.1021/ACS.JPCC.8B00388.

(4) Umadevi, S.; Lee, H. C.; Ganesh, V.; Feng, X.; Hegmann, T. A Versatile, One-Pot Synthesis of Gold Nanostars with Long, Well-Defined Thorns Using a Lyotropic Liquid Crystal Template. *Liq. Cryst.* **2014**, *41* (3), 265–276. https://doi.org/10.1080/02678292.2013.798691.

(5) Bazán-Díaz, L.; Mendoza-Cruz, R.; Velázquez-Salazar, J. J.; Plascencia-Villa, G.; Romeu, D.; Reyes-Gasga, J.; Herrera-Becerra, R.; José-Yacamán, M.; Guisbiers, G. Gold–Copper Nanostars as Photo-Thermal Agents: Synthesis and Advanced Electron Microscopy Characterization. *Nanoscale* **2015**, *7* (48), 20734–20742. https://doi.org/10.1039/C5NR06491K.

(6) Theodorou, I. G.; Jawad, Z. A. R.; Jiang, Q.; Aboagye, E. O.; Porter, A. E.; Ryan, M. P.; Xie, F. Gold Nanostar Substrates for Metal-Enhanced Fluorescence through the First and Second Near-Infrared Windows. *Chem. Mater.* **2017**, *29* (16), 6916–6926. https://doi.org/10.1021/acs.chemmater.7b02313.

(7) Atta, S.; Beetz, M.; Fabris, L. Understanding the Role of AgNO3 Concentration and Seed Morphology in the Achievement of Tunable Shape Control in Gold Nanostars. *Nanoscale* **2019**, *11* (6), 2946–2958. https://doi.org/10.1039/C8NR07615D.

(8) Chandra, K.; Culver, S. B. K.; Werner, S. E.; Lee, R. C.; Odom, T. W. Manipulating the Anisotropic Structure of Gold Nanostars Using Good's Buffers. *Chem. Mater.* **2016**, *28* (18), 6763–6769. https://doi.org/10.1021/acs.chemmater.6b03242.

(9) Khoury, C. G.; Vo-Dinh, T. Gold Nanostars for Surface-Enhanced Raman Scattering: Synthesis, Characterization and Optimization. *J. Phys. Chem. C* **2008**, *112* (48), 18849–18859. https://doi.org/10.1021/JP8054747.

(10) Sohrabi Kashani, A.; Badilescu, S.; Piekny, A.; Packirisamy, M. Differing Affinities of Gold Nanostars and Nanospheres toward HeLa and HepG2 Cells: Implications for Cancer Therapy. *ACS Appl. Nano Mater.* **2020**, *3* (5), 4114–4126. https://doi.org/10.1021/acsanm.0c00244.

(11) Pallares, R. M.; Stilson, T.; Choo, P.; Hu, J.; Odom, T. W. Using Good's Buffers to Control the Anisotropic Structure and Optical Properties of Spiky Gold Nanoparticles for Refractive Index Sensing. *ACS Appl. Nano Mater.* **2019**, *2* (8), 5266–5271. https://doi.org/10.1021/ACSANM.9B01117.

(12) Xu, P.; Feng, Q.; Yang, X.; Liu, S.; Xu, C.; Huang, L.; Chen, M.; Liang, F.; Cheng, Y. Near Infrared Light Triggered Cucurbit[7]Uril-Stabilized Gold Nanostars as a Supramolecular Nanoplatform for Combination Treatment of Cancer. *Bioconjug. Chem.* **2018**, *29* (8), 2855–2866. https://doi.org/10.1021/acs.bioconjchem.8b00438.

(13) Cai, Z.; Zhang, Y.; He, Z.; Jiang, L.-P.; Zhu, J.-J. NIR-Triggered Chemo-Photothermal Therapy by Thermosensitive Gold Nanostar@Mesoporous Silica@Liposome-Composited Drug Delivery Systems. *ACS Appl. Bio Mater.* **2020**, *3* (8), 5322–5330. https://doi.org/10.1021/acsabm.0c00651.

(14) Harmsen, S.; Huang, R.; Wall, M. A.; Karabeber, H.; Samii, J. M.; Spaliviero, M.; White, J. R.; Monette, S.; O'Connor, R.; Pitter, K. L.; Sastra, S. A.; Saborowski, M.; Holland, E. C.; Singer, S.; Olive, K. P.; Lowe, S. W.; Blasberg, R. G.; Kircher, M. F. Surface-Enhanced Resonance Raman Scattering Nanostars for High Precision Cancer Imaging. *Sci. Transl. Med.* **2015**, *7* (271), 271ra7.

https://doi.org/10.1126/SCITRANSLMED.3010633.
(15) M. Fales, A.; Yuan, H.; Vo-Dinh, T. Development of Hybrid Silver-Coated Gold Nanostars for Nonaggregated Surface-Enhanced Raman Scattering. *J. Phys. Chem. C* **2014**, *118* (7), 3708–3715. https://doi.org/10.1021/jp4091393.
(16) Maiorano, G.; Rizzello, L.; Malvindi, M. A.; Shankar, S. S.; Martiradonna, L.; Falqui, A.; Cingolani, R.; Pompa, P. P. Monodispersed and Size-Controlled Multibranched Gold Nanoparticles with Nanoscale Tuning of Surface Morphology. *Nanoscale* **2011**, *3* (5), 2227–2232. https://doi.org/10.1039/C1NR10107B.
(17) Wang, S.-S.; Zhao, X.-P.; Liu, F.-F.; Rizwan Younis, M.; Xia, X.-H.; Wang, C. Direct Plasmon-Enhanced Electrochemistry for Enabling Ultrasensitive and Label-Free Detection of Circulating Tumor Cells in Blood. *Anal. Chem.* **2019**, *91* (7), 4413–4420. https://doi.org/10.1021/acs.analchem.8b04908.
(18) Jimenez de Aberasturi, D.; B. Serrano-Montes, A.; Langer, J.; Henriksen-Lacey, M.; J. Parak, W.; M. Liz-Marzán, L. Surface Enhanced Raman Scattering Encoded Gold Nanostars for Multiplexed Cell Discrimination. *Chem. Mater.* **2016**, *28* (18), 6779–6790. https://doi.org/10.1021/acs.chemmater.6b03349.
(19) Yuan, H.; Khoury, C. G.; Hwang, H.; Wilson, C. M.; Grant, G. A.; Vo-Dinh, T. Gold Nanostars: Surfactant-Free Synthesis, 3D Modelling, and Two-Photon Photoluminescence Imaging. *Nanotechnology* **2012**, *23* (7), 075102. https://doi.org/10.1088/0957-4484/23/7/075102.
(20) Li, L.; Liao, M.; Chen, Y.; Shan, B.; Li, M. Surface-Enhanced Raman Spectroscopy (SERS) Nanoprobes for Ratiometric Detection of Cancer Cells. *J. Mater. Chem. B* **2019**, *7* (5), 815–822. https://doi.org/10.1039/C8TB02828A.
(21) Pan, Y.; Ma, X.; Liu, C.; Xing, J.; Zhou, S.; Parshad, B.; Schwerdtle, T.; Li, W.; Wu, A.; Haag, R. Retinoic Acid-Loaded Dendritic Polyglycerol-Conjugated Gold Nanostars for Targeted Photothermal Therapy in Breast Cancer Stem Cells. *ACS Nano* **2021**, *15* (9), 15069–15084. https://doi.org/10.1021/acsnano.1c05452.
(22) Lu, G.; Forbes, T. Z.; Haes, A. J. SERS Detection of Uranyl Using Functionalized Gold Nanostars Promoted by Nanoparticle Shape and Size. *Analyst* **2016**, *141* (17), 5137–5143. https://doi.org/10.1039/C6AN00891G.
(23) Titian Ginting, R.; Kaur, S.; Lim, D.-K.; Kim, J.-M.; Hee Lee, J.; Hee Lee, S.; Kang, J.-W. Plasmonic Effect of Gold Nanostars in Highly Efficient Organic and Perovskite Solar Cells. *ACS Appl. Mater. & Interfaces* **2017**, *9* (41), 36111–36118. https://doi.org/10.1021/acsami.7b11084.
(24) Pramanik, A.; Mayer, J.; Patibandla, S.; Gates, K.; Gao, Y.; Davis, D.; Seshadri, R.; Chandra Ray, P. Mixed-Dimensional Heterostructure Material-Based SERS for Trace Level Identification of Breast Cancer-Derived Exosomes. *ACS Omega* **2020**, *5* (27), 16602–16611. https://doi.org/10.1021/acsomega.0c01441.
(25) Batmunkh, M.; Macdonald, T. J.; Peveler, W. J.; Bati, A. S. R.; Carmalt, C. J.; Parkin, I. P.; Shapter, J. G. Plasmonic Gold Nanostars Incorporated into High-Efficiency Perovskite Solar Cells. *ChemSusChem* **2017**, *10* (19), 3750–3753. https://doi.org/10.1002/CSSC.201701056.
(26) Barbosa, S.; Agrawal, A.; Rodríguez-Lorenzo, L.; Pastoriza-Santos, I.; Alvarez-Puebla, R. A.; Kornowski, A.; Weller, H.; Liz-Marzán, L. M. Tuning Size and Sensing Properties in Colloidal Gold Nanostars. *Langmuir* **2010**, *26* (18), 14943–14950. https://doi.org/10.1021/la102559e.
(27) Hu, Y.; Liu, Y.; Xie, X.; Bao, W.; Hao, J. Surfactant-Regulated Fabrication of Gold Nanostars in Magnetic Core/Shell Hybrid Nanoparticles for Controlled Release of Drug. *J. Colloid Interface Sci.* **2018**, *529*, 547–555. https://doi.org/10.1016/J.JCIS.2018.06.027.

(28) Nehl, C. L.; Liao, H.; Hafner, J. H. Optical Properties of Star-Shaped Gold Nanoparticles. *Nano Lett.* **2006**, *6* (4), 683–688. https://doi.org/10.1021/nl052409y.
(29) Silva, A. G. M. da; Rodrigues, T. S.; Macedo, A.; Silva, R. T. P. da; Camargo, P. H. C. An Undergraduate Level Experiment on the Synthesis of Au Nanoparticles and Their Size-Dependent Optical and Catalytic Properties. *Quim. Nova* **2014**, *37* (10), 1716–1720. https://doi.org/10.5935/0100-4042.20140235.
(30) Ma, T.; Yang, W.; Liu, S.; Zhang, H.; Liang, F. A Comparison Reduction of 4-Nitrophenol by Gold Nanospheres and Gold Nanostars. *Catal. 2017, Vol. 7, Page 38* **2017**, *7* (2), 38. https://doi.org/10.3390/CATAL7020038.
(31) Ndokoye, P.; Zhao, Q.; Li, X.; Li, T.; Tade, M. O.; Wang, S. Branch Number Matters: Promoting Catalytic Reduction of 4-Nitrophenol over Gold Nanostars by Raising the Number of Branches and Coating with Mesoporous SiO2. *J. Colloid Interface Sci.* **2016**, *477*, 1–7. https://doi.org/10.1016/J.JCIS.2015.11.032.
(32) Deshmukh, A. R.; Kim, B. S. Flower-like Biogenic Gold Nanostructures for Improved Catalytic Reduction of 4-Nitrophenol. *J. Environ. Chem. Eng.* **2021**, *9* (6), 106707. https://doi.org/10.1016/J.JECE.2021.106707.
(33) Ma, H.; Liu, Z.; Wei, Y.; Jiang, L. Controlled Morphology Evolution of Branched Au Nanostructures and Their Shape-Dependent Catalytic and Photo-Thermal Properties. *Colloids Surfaces A Physicochem. Eng. Asp.* **2019**, *582*, 123889. https://doi.org/10.1016/J.COLSURFA.2019.123889.
(34) Nehra, K.; Kumar Pandian, S.; Byram, C.; Satya Bharati Moram, S.; Rao Soma, V. Quantitative Analysis of Catalysis and SERS Performance in Hollow and Star-Shaped Au Nanostructures. *J. Phys. Chem. C* **2019**, *123* (26), 16210–16222. https://doi.org/10.1021/acs.jpcc.9b03086.
(35) Li, F.; Li, Z.; Zeng, C.; Hu, Y. Laccase-Assisted Rapid Synthesis of Colloidal Gold Nanoparticles for the Catalytic Reduction of 4-Nitrophenol. *J. Braz. Chem. Soc.* **2017**, *28* (6), 960–966. https://doi.org/10.21577/0103-5053.20160246.
(36) Liu, Y.; Zhang, Y.; Tardivel, M.; Lequeux, M.; Chen, X.; Liu, W.; Huang, J.; Tian, H.; Liu, Q.; Huang, G.; Gillibert, R.; de la Chapelle, M. L.; Fu, W. Evaluation of the Reliability of Six Commercial SERS Substrates. *Plasmonics* **2020**, *15* (3), 743–752. https://doi.org/10.1007/S11468-019-01084-8.
(37) Shen, Y.; Jing, X.; Mu, X.; -, al; Chiter, F.; Costa, D.; Maurice, V.; Ma, W.; Fang, Y.; Hao, G.; Wang, W. Adsorption Behaviors of 4-Mercaptobenzoic Acid on Silver and Gold Films. *Chinese J. Chem. Phys.* **2010**, *23* (6), 659. https://doi.org/10.1088/1674-0068/23/06/659-663.
(38) Anh, M. N. T.; Nguyen, D. T. D.; Ching, Y. C.; Thanh, N. V. K.; Phong, N. T. P.; Nguyen, D. H.; Bui, B. T.; Nguyen-Le, M. T. Unveiling the SERS Activity of Silver Triangular Nanoplates in the Enhanced Detection of 4-Mercaptobenzoic Acid. *Optik (Stuttg).* **2021**, *248*, 168155. https://doi.org/10.1016/J.IJLEO.2021.168155.
(39) Bullen, C.; Zijlstra, P.; Bakker, E.; Gu, M.; Raston, C. Chemical Kinetics of Gold Nanorod Growth in Aqueous CTAB Solutions. *Cryst. Growth Des.* **2011**, *11* (8), 3375–3380. https://doi.org/10.1021/cg101636r.
(40) Chen, Y. C.; Chen, J. Y.; Wu, W. W. In Situ Observation of Au Nanostructure Evolution in Liquid Cell TEM. *J. Phys. Chem. C* **2017**, *121* (46), 26069–26075. https://doi.org/10.1021/ACS.JPCC.7B07956.
(41) Karimadom, B. R.; Kornweitz, H. Mechanism of Producing Metallic Nanoparticles, with an Emphasis on Silver and Gold Nanoparticles, Using Bottom-Up Methods. *Molecules* **2021**, *26* (10), 2968. https://doi.org/10.3390/MOLECULES26102968.
(42) *Electrochemical Series*; ASM International, 2005. https://doi.org/10.31399/ASM.HB.V13B.A0006542.

(43) Miranda, M. A. R.; Sasaki, J. M. The Limit of Application of the Scherrer Equation. *Acta Crystallogr. Sect. A Found. Adv.* **2018**, *74* (1), 54–65. https://doi.org/10.1107/S2053273317014929.

(44) Litti, L.; Meneghetti, M. Predictions on the SERS Enhancement Factor of Gold Nanosphere Aggregate Samples. *Phys. Chem. Chem. Phys.* **2019**, *21* (28), 15515–15522. https://doi.org/10.1039/C9CP02015B.

(45) Le Ru, E. C.; Blackie, E.; Meyer, M.; Etchegoint, P. G. Surface Enhanced Raman Scattering Enhancement Factors: A Comprehensive Study. *J. Phys. Chem. C* **2007**, *111* (37), 13794–13803. https://doi.org/10.1021/JP0687908/SUPPL_FILE/JP0687908SI20070717_104641.PDF.

(46) Drechsler, A.; Lieb, M. A.; Debus, C.; Meixner, A. J.; Tarrach, G.; R Sheppard, C. J.; Choudhury, A.; Gannaway, J. Confocal Microscopy with a High Numerical Aperture Parabolic Mirror. *Opt. Express, Vol. 9, Issue 12, pp. 637-644* **2001**, *9* (12), 637–644. https://doi.org/10.1364/OE.9.000637.
